# Dynamic changes in immune cell populations by AXL kinase targeting diminish liver inflammation and fibrosis in experimental MASH

**DOI:** 10.3389/fimmu.2024.1400553

**Published:** 2024-05-16

**Authors:** Sturla Magnus Grøndal, Anna Tutusaus, Loreto Boix, Maria Reig, Magnus Blø, Linn Hodneland, Gro Gausdal, Akil Jackson, Pablo Garcia de Frutos, James Bradley Lorens, Albert Morales, Montserrat Marí

**Affiliations:** ^1^ Department of Biomedicine, Centre for Cancer Biomarkers, University of Bergen, Bergen, Norway; ^2^ Institute of Biomedical Research of Barcelona (IIBB-CSIC), Institut d'Investigacions Biomèdiques August Pi i Sunyer (IDIBAPS), Barcelona, Spain; ^3^ Barcelona Clinic Liver Cancer Center (BCLC), Hospital Clínic de Barcelona, Universitat de Barcelona, Institut d'Investigacions Biomèdiques August Pi i Sunyer (IDIBAPS), Centro de Investigación Biomédica en Red de Enfermedades Hepáticas y Digestivas (CIBEREHD), Barcelona, Spain; ^4^ BerGenBio ASA, Bergen, Norway; ^5^ BerGenBio Ltd., Oxford, United Kingdom; ^6^ Unidad Asociada (IMIM), Institute of Biomedical Research of Barcelona (IIBB-CSIC), Barcelona, Spain; ^7^ Centro de Investigación Biomédica en Red de Enfermedades Cardiovasculares (CIBERCV), ISCIII, Madrid, Spain

**Keywords:** TAM receptors, mass cytometry, MERTK, GAS6, liver fibrosis, inflammation, immune response

## Abstract

**Background and aims:**

Metabolic dysfunction-associated steatohepatitis (MASH) is a significant health concern with limited treatment options. AXL, a receptor tyrosine kinase activated by the GAS6 ligand, promotes MASH through activation of hepatic stellate cells and inflammatory macrophages. This study identified cell subsets affected by MASH progression and the effect of AXL inhibition.

**Methods:**

Mice were fed chow or different fat-enriched diets to induce MASH, and small molecule AXL kinase inhibition with bemcentinib was evaluated. Gene expression was measured by qPCR. Time-of-flight mass cytometry (CyTOF) used single cells from dissociated livers, acquired on the Fluidigm Helios, and cell populations were studied using machine learning.

**Results:**

In mice fed different fat-enriched diets, liver steatosis alone was insufficient to elevate plasma soluble AXL (sAXL) levels. However, in conjunction with inflammation, sAXL increases, serving as an early indicator of steatohepatitis progression. Bemcentinib, an AXL inhibitor, effectively reduced proinflammatory responses in MASH models, even before fibrosis appearance. Utilizing CyTOF analysis, we detected a decreased population of Kupffer cells during MASH while promoting infiltration of monocytes/macrophages and CD8^+^ T cells. Bemcentinib partially restored Kupffer cells, reduced pDCs and GzmB^−^ NK cells, and increased GzmB^+^CD8^+^ T cells and LSECs. Additionally, AXL inhibition enhanced a subtype of GzmB^+^CD8^+^ tissue-resident memory T cells characterized by CX3CR1 expression. Furthermore, bemcentinib altered the transcriptomic landscape associated with MASH progression, particularly in TLR signaling and inflammatory response, exhibiting differential cytokine expression in the plasma, consistent with liver repair and decreased inflammation.

**Conclusion:**

Our findings highlight sAXL as a biomarker for monitoring MASH progression and demonstrate that AXL targeting shifted liver macrophages and CD8^+^ T-cell subsets away from an inflammatory phenotype toward fibrotic resolution and organ healing, presenting a promising strategy for MASH treatment.

## 1 Introduction

Metabolic dysfunction-associated steatohepatitis (MASH) has emerged as a significant health concern worldwide, characterized by hepatic steatosis, inflammation, and progressive fibrosis. It is an advanced stage of metabolic dysfunction-associated steatotic liver disease (MASLD), previously known as non-alcoholic fatty liver disease (NAFLD), and has the potential to progress to cirrhosis and liver cancer. The pathogenesis of MASH involves complex interactions between metabolic dysregulation, oxidative stress, lipid accumulation, and immune-mediated inflammation within the liver (1–3).

Recent studies demonstrate the role of receptor tyrosine kinases (RTKs) in the pathogenesis of MASH (4, 5) and its role in liver fibrosis (6–8). Among these, AXL, a member of the TAM (TYRO3, AXL, and MerTK) family of RTKs, has frequently been linked to inflammatory processes.

AXL activation is dependent on its γ-carboxylated ligand growth arrest-specific 6 (GAS6), which acts as a bridging molecule to externalized phosphatidylserine (9). AXL has been implicated in various cellular processes, including cell survival, proliferation, migration, and inflammation (10). Its involvement in tumorigenesis and autoimmune diseases has been extensively studied (11–13); however, in liver diseases, particularly in MASH, research on the role of AXL is limited (4, 7, 8, 14–16).

Previously, AXL was shown to be expressed by hepatic stellate cells (HSCs) and induce profibrotic signaling (7). AXL has also been shown to be expressed by Kupffer cells and macrophages, contributing to hepatic fibrogenesis, inflammation and, ultimately, liver damage (4). Moreover, AXL-mediated signaling pathways have been implicated in the innate and adaptive immune responses, altered during MASH (10, 12, 13), suggesting its potential as a therapeutic target.

In this study, we aimed to investigate the significance of AXL expression at the cellular level in the progression of MASH and to evaluate the effects of AXL inhibition on liver inflammation and fibrosis. To accomplish this, we utilized mouse models fed different fat-enriched diets to induce a MASH-like pathology and to ascertain when GAS6/AXL signaling is activated and is relevant as a biomarker. We also evaluated if AXL inhibition is effective in early MASLD treatment when soluble AXL (sAXL) serum levels increase. To gain insights into the cellular and molecular mechanisms underlying the protective effects of AXL inhibition during MASH pathogenesis, we employed cytometry by time-of-flight (CyTOF), also known as mass cytometry, and transcriptomic analysis on liver samples from mice fed with MASH-promoting diet and/or the AXL inhibitor bemcentinib.

## 2 Materials and methods

### 2.1 Animal care and *in vivo* models

Animal studies were approved by the institutional animal care committee (Universitat de Barcelona). Male, 8-week-old C57BL/6J mice were bought from Charles River. All mice were maintained with a 12-h light/dark cycle (lights on at 8:00 a.m.) in a temperature-controlled environment. To induce MASLD-MASH, mice were placed on either a high-fructose, high-fat diet (HFHF, D12492, Research diets + 15% fructose in drinking water) with 60% kcal coming from fat (lard and soybean oil) for 3 months or a high-fat diet (HFD, #A06071302, Research diets) with 60% kcal from fat (lard and soybean oil), with 0.1% methionine and no added choline, for 2, 4, or 8 weeks. To assess the effect of AXL inhibition, daily doses of bemcentinib (0–100 mg/kg) or vehicle were given, while continuing on HFD, by oral gavage for the last 1 or 2 weeks, depending on the length of the study. Also, B6/129 male mice (8–12 weeks of age) were fed *ad libitum* for 1 year a high-fat, high-carbohydrate diet (Western diet, WD) with 42% kcal from fat and containing 0.1% cholesterol (Harlan TD.88137) with a high fructose–glucose solution (SW, 23.1 g/L of d-fructose + 18.9 g/L of d-glucose), as previously described (17). These mice develop obesity, liver injury, dyslipidemia, and insulin resistance. Control mice were fed a standard chow diet (CD, Harlan TD.7012) with normal water.

### 2.2 Alanine and aspartate transaminases

ALT and AST in serum samples from treated mice and triglycerides from mouse liver extracts were measured using biochemical analyzers at the Clinic Hospital Core in Barcelona.

### 2.3 H&E and Sirius Red staining

Livers were formalin-fixed and 7-μm sections were routinely stained with H&E or a 0.1% Sirius Red-picric solution following standard procedures. The slices were examined with a Nikon Eclipse E-1000 microscope equipped with an Olympus DP72 camera. For collagen–fiber determination, a series of six random-selected fields from each slice were visualized and quantified using ImageJ software.

### 2.4 NAFLD activity score

NAFLD activity score (NAS) score was determined in H&E samples as previously reported (18). In brief, NAS was assessed blindly, evaluating the degree of steatosis (0–3), lobular inflammation (0–3), and ballooning (0–2). According to this algorithm, MASLD requires the presence of steatosis in >5% of hepatocytes, as well as MASH, in addition to steatosis, of hepatocellular ballooning of any degree and focus of inflammatory cells within the lobule.

### 2.5 Determination of soluble AXL levels

sAXL levels were determined in mouse serum samples by specific sandwich ELISA using commercial kits (DY854, mouse AXL DuoSet ELISA, R&D, Minneapolis, MN, USA) following the manufacturer’s instructions.

### 2.6 RNA isolation and real-time PCR

Total hepatic RNA was isolated with a TRIzol reagent. RNA (1 µg) was reverse-transcribed with iScript™ cDNA Synthesis Kit (Bio-Rad Laboratories), and real-time PCR was performed with iTaq™ Universal SYBR^®^ Green Supermix (Bio-Rad Laboratories) following the manufacturer’s instructions. The expression levels of selected genes were normalized to that of the β-actin gene. Fold change gene expression was calculated by the 2^−ΔΔCt^ method normalizing against average values of control mice fed with a standard chow diet. The primer sequences used were as follows:

**Table:** 

	Forward primer	Reverse primer
Mouse Acta2	ATG GCT CTG GGC TCT GTA AG	CCC ATT CCA ACC ATT ACT CC
Mouse Col1A1	GAG CGG AGA GTA CTG GAT CG	GTT CGG GCT GAT GTA CCA GT
Mouse Col6A3	ACA GGC AAA GCC CTC AAC CT	AAC CAG CAGC ACC AGG AAC T
Mouse MMP2	ACC TGA AGC TGG AGA ACC AA	CAC ATC CTT CAC CTG GTG TG
Mouse CCR2	ATC CAC GGC ATA CTA TCA ACA TC	CAA GGC TCA CCA TCA TCG TAG
Mouse MPO	TGC TGA AGA ACC TGG AGT TG	AAA CCG ATC ACC ATC ACG TA
Mouse TNF	CTG AAC TTC GGG GTG ATC GGT	ACG TGG GCT ACA GGC TTG TCA
Mouse MCP1	CAA GAA GGA ATG GGT CCA GA	GCT GAA GAC CTT AGG GCA GA
Mouse β-actin	GAC GGC CAG GTC ATC ACT AT	CGG ATG TCA ACG TCA CAC TT

### 2.7 mRNA array for innate and adaptive immune responses

Quantitative real-time PCR (qPCR) was done using PrimePCR arrays (Bio-Rad # 10034352, Hercules, CA, USA) containing the genes of interest. In brief, total RNA (500 ng) was converted to cDNA using the iScript cDNA synthesis kit (Bio-Rad, Hercules, CA, USA). The generated cDNA was mixed with SsoAdvanced SYBR green 2X master mix and loaded (20 μL/per well) on a customized PrimePCR plate containing primers for selected genes to be validated including housekeeping genes and controls as described (www.bio-rad.com/PrimePCR). The PCR amplification was carried out on the CFX384™ Real-time PCR detection system (Bio-Rad Laboratories) as described by the manufacturer.

### 2.8 Cytokine array

Mouse Cytokine Array (GSM-CYT-1, RayBiotech, Atlanta, GA, USA) was used for the measurement of 20 mouse cytokines. Serum samples from all mice groups were hybridized according to the manufacturer’s instructions and analyzed using an InnoScan 710 laser scanner for glass slides, and the Mapix software was used for quantifications.

### 2.9 Tissue digestion protocol for CYTOF

Mice were anesthetized with sodium pentobarbital (50 mg/kg), and after collecting a blood sample, the liver was harvested and placed in a 15-mL tube containing 3 mL of tissue storage medium (cat# 130–100-008, Miltenyi Biotec, Bergisch Gladbach, Germany) on ice. The dissociation was performed with the Multi Tissue Dissociation Kit (#130–110-201, Miltenyi Biotec) following the manufacturer’s specifications, using the right liver lobule and 5 mL of working volume. Once red blood cells were removed, the cell pellet was resuspended in 500 µl of RPMI before adding 500 µl of cisplatin/rhodium intercalator solution [10 µM of cisplatin (natural abundance), 4 µM of Rhodium intercalator (cat# 201103A, Fluidigm, San Francisco, CA, USA) in RPMI 1640] for live/dead discrimination for 5 min RT before being quenched by 5 mL of RPMI 1640 (10% FBS, 1% DNase). Finally, cells were fixed in RPMI 1640 with 1.6% PFA for 10 min and transferred to cryotubes. The pellets were stored at −80°C.

### 2.10 Cell counting

Thawed cells were resuspended in DPBS (0.25 mg/mL of DNase, 1% BSA), and the concentration was determined using Cell Countess. Cells were aliquoted and centrifuged, and the pellet was resuspended in FBS (10% DMSO) before freezing at −80°C.

### 2.11 Barcoding

Thawed cells were washed with DPBS (0.25 mg/mL of DNase, 1% BSA) and then resuspended in barcode perm buffer and barcoded according to the manufacturer’s instructions (cat# 201060, Fluidigm). After barcoding, cells were washed and pooled in FBS (10% DMSO), aliquoted, and stored at −80°C. For cisplatin barcoding, cells were incubated with 25 nM of monoisotopic cisplatin (Pt196, Pt198), quenched with 1 M of Tris, washed, and stored at −80°C.

### 2.12 Antibody conjugation

Antibody conjugation with 157Gd was performed according to the manufacturer’s instructions (Ionpath Inc., Menlo Park, CA, USA). Cadmium-labeled antibodies were conjugated according to the cadmium protocol, and lanthanide-labeled antibodies were conjugated according to the lanthanide protocol provided by the manufacturer (Fluidigm). For conjugation of indium-labeled antibodies, metal salts were dissolved in L-buffer (Maxpar^®^ X8 Antibody Labeling Kit, Fluidigm) to yield a stock solution of 1,000 mM. A diluted solution of 50 mM of metal solution was used for antibody conjugation according to the lanthanide protocol.

### 2.13 Mass cytometry staining

Barcoded cells were thawed, washed, and blocked (DPBS; 0.5% BSA; 2 µg/mL of antibody clones 9e9, 93, and 2.4G2; 1 kU/mL of heparin). The surface antibody cocktail was added and cells were incubated at 4°C overnight on a rotisserie. The next day, cells were washed, fixed in 2% PFA for 5 min RT, and then quenched with 1 M of Tris. The cells were then permeabilized in −20°C MeOH for 10 min on ice. Next, the cells were washed, blocked as previously described, and stained with the intracellular cocktail overnight at 4°C. The next day, cells were washed and resuspended in PBS (2 mM of EDTA, 0.1 μM of iridium intercalator, 2% PFA) and incubated at 4°C overnight. The next day, the fixative was quenched with 1 M of Tris, and cells were washed in DPBS (0.5% BSA, 0.25 mg/mL of DNase), followed by washing in PBS (2 mM of EDTA). Aliquots of cells were washed in Milli-Q immediately before running and acquired in Milli-Q with EQ beads.

### 2.14 Data analysis

IMD files were converted to FCS files using default settings in CyTOF (version 6.7.1014, Fluidigm). Subsequent analyses were conducted in R (version 4.1.3). FCS files were read (FlowCore) and normalized (premessa R package version 0.2.6). XML gating files, generated in FlowJo (version 10.7.1), facilitated automatic gating through flowUtils. Concatenated gated files underwent debarcoding (CATALYST) (19) and spillover compensation (CATALYST) using a pre-established spillover matrix. Data transformation was performed using an inverse hyperbolic sine function with a cofactor of 5. To correct for signal variability, the transformed data for each marker were normalized against the iridium signal. UMAP embeddings (uwot) and clustering (parc) (20) were conducted using a cosine similarity metric. Clusters identified as debris were excluded and the remaining clusters were curated manually. The final dataset comprised 5,365,781 events. UMAPs and heatmaps were generated using the R packages ggplot2 and heatmaply.

See **Supplementary Methods** for more detailed information.

### 2.15 Statistical analysis

Results are expressed as median and interquartile range (IQR), unless otherwise specified. Non-parametric Mann–Whitney test or Kruskal–Wallis followed by Dunn multiple comparisons was performed to determine statistical significance. All analyses were performed using GraphPad Prism. Differences were considered significant when *p <*0.05. The coefficient of determination (*r*) and statistical significance (*p*-value) were determined using Pearson’s or Spearman’s coefficients. For CyTOF data, cluster compositions were analyzed using the acomp function in the compositions R package, acknowledging the closed compositional nature of the data. Transformations were applied using the clr function. Proportionality between cluster sizes was performed using rho in the propr R package. To test if treatment groups were different, 100,000 Monte Carlo replicates of the Dirichlet distribution using the ALDEx2 R package (21) were performed, followed by the correction of multiple hypothesis testing with the Benjamini–Hochberg procedure.

## 3 Results

### 3.1 Increased serum sAXL is associated with active inflammation in MASLD-afflicted mice and predicts fibrosis

**Figure 1:**
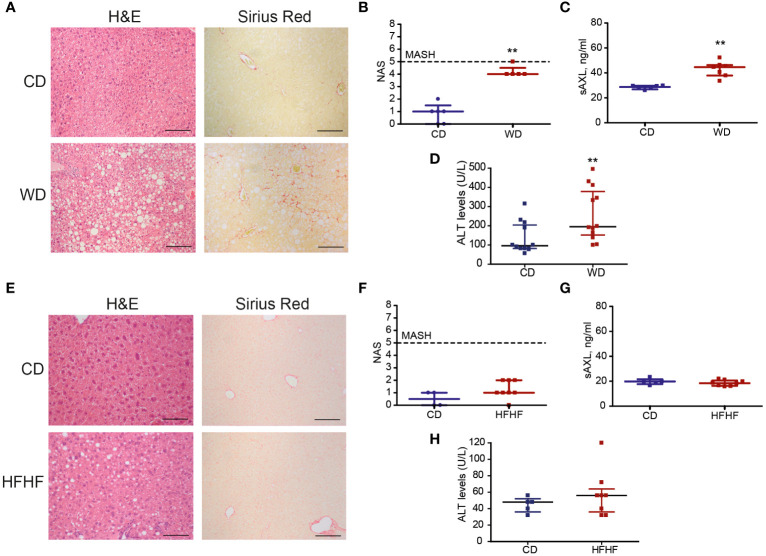
Soluble AXL (sAXL) increases in the sera of steatotic mice before the onset of histological MASH. Representative images of liver sections after H&E and Sirius Red staining **(A, E)** non-alcoholic fatty liver disease (NAFLD) activity score (NAS) **(B, F)**, ALT levels **(C, G)**, and serum sAXL levels measured by ELISA **(D, H)** of mice fed with Western diet (WD) plus glucose/sucrose solution for 1 year (*n* = 5) and mice fed with a high-fat with fructose (HFHF) diet for 3 months (*n* = 8) compared with the respective control diet (CD) fed mice (*n* = 6, *n* = 4). Scale bar, 200 μm. H&E, hematoxylin and eosin. Mann–Whitney test; **p < 0.01 vs. CD.

Our previous results identified the GAS6/TAM pathway as a relevant mechanism activated in patients suffering from MASH, which could be prevented by targeting AXL as demonstrated in experimental animal models of fibrosis and MASH (4). In particular, sAXL was markedly increased in mice fed for 8 weeks with a high-fat, choline-deficient, and methionine-restricted (HFD) diet. To verify if sAXL changes are common to other MASLD conditions, we used the Western diet (WD) and high fat with high fructose (HFF) animal models. Initially, we measured sAXL in animals fed with WD plus glucose/sucrose solution for 1 year. Mice subjected to this diet developed obesity, liver injury, dyslipidemia, and insulin resistance, as frequently exhibited in MASH development (17). Our results demonstrated signs of liver steatosis and fibrosis in WD-treated mice (**Figure 1A**), with indications of inflammation reflected in the MASLD activity score (NAS) (**Figure 1B**). In this WD model displaying simultaneously steatosis, fibrosis, inflammation, and liver damage (**Figure 1C**), we detected a marked increase in serum sAXL after WD feeding compared with control mice (**Figure 1D**). In contrast, mice fed HFF for 3 months developed liver steatosis without signs of fibrosis in the liver parenchyma, as reflected by H&E and Sirius Red staining (**Figure 1E**). Moreover, no major change in liver inflammation was observed, as illustrated by NAS (**Figure 1F**), or hepatic damage (**Figure 1G**), and reflected by the lack of significant mRNA changes in inflammatory genes from HFF-treated mice (data not shown). In the HFF mice model, which did not have fibrosis and inflammation, we found no increase in sAXL levels despite the presence of liver steatosis (**Figure 1H**). Therefore, steatosis without concurrent development of fibrosis seems to be insufficient to elevate serum sAXL in the HFF model where liver inflammation is not detected. In contrast, WD, which displayed fatty livers with significant fibrosis and inflammation, showed a significant increase in serum sAXL.

To better understand the timing and influence of serum sAXL elevation in MASLD during the onset of liver fibrosis and inflammation, we employed the same HFD used in our previous MASH studies (4) and measured serum sAXL after 2 and 4 weeks on HFD.

**Figure 2:**
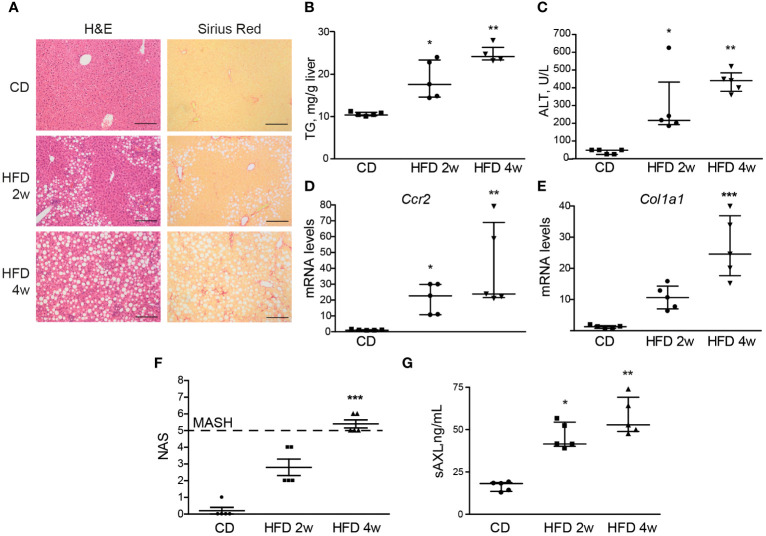
Serum sAXL increases in concert with MASLD progression. Liver sections of mice fed with a high-fat, choline-deficient, and methionine-restricted diet (HFD) for 2 and 4 weeks were stained with H&E and Sirius Red **(A)**. Triglycerides in liver extracts **(B)** and serum alanine aminotransferase (ALT) transaminases **(C)** were measured. mRNA expression level of Ccr2 and Col1a1 was measured in the liver samples **(D, E)**. NAS was assessed **(F)**, and serum sAXL was quantified by ELISA **(G)**. Scale bar, 200 μm. H&E, hematoxylin and eosin; NAS, non-alcoholic fatty liver disease activity score. Kruskal–Wallis followed by Dunn multiple comparisons; *p ≤ 0.05, **p ≤ 0.01, and ***p ≤ 0.001 vs. control diet (CD), *n* = 5.

After 4 weeks of HFD feeding, liver steatosis, ballooning, and fibrosis were detected in the mice (**Figure 2A**). We also found increased levels of triglycerides, alanine aminotransferase (ALT), *Ccr2*, and *Col1a1* mRNAs, which indicate steatosis, hepatocellular damage, liver inflammation, and collagen deposition. In combination, these provide evidence of early MASH (**Figures 2B–F**). Interestingly, after 2 weeks of feeding, mice presented with fatty livers in the absence of significant liver fibrosis as measured by Sirius Red staining (**Figure 2A**), although transcriptomic alteration on fibrogenic genes was already elevated (**Figure 2E**). In contrast to the previously studied HFF steatotic model, 2-week HFD-fed mice exhibited signs of inflammation in the liver parenchyma as quantified with NAS (**Figure 2F**). Of note, both groups showed a significant increase in serum sAXL, suggesting that AXL plays a role prior to the appearance of detectable histological fibrosis and as soon as evidence of liver inflammation is detectable in the fatty liver (**Figure 2G**).

### 3.2 AXL inhibition ameliorates liver steatosis, fibrosis, and inflammation in early MASH

We have previously demonstrated that bemcentinib, a specific AXL kinase inhibitor, effectively reduces liver fibrosis and inflammation in mice (4). These mice were fed HFD for 8 weeks and treated with bemcentinib, while on HFD, during the final 2 weeks, representing advanced-stage MASH. Since our present results reveal the presence of serum sAXL as an early marker of MASLD, even before fibrosis detection, we wanted to evaluate if AXL inhibition could be effective in protecting the liver as soon as serum sAXL started increasing. Therefore, we placed mice on HFD for 4 weeks and started treatment with bemcentinib after 2 weeks, while on HFD. In this setting, AXL inhibition reduced liver steatosis and fibrosis, as observed with H&E and Sirius Red staining (**Supplementary Figure 1**). Liver triglyceride content was diminished as well as inflammation, as indicated by reduced *Ccr2* mRNA levels and minor lobular inflammation in bemcentinib-treated mice, resulting in a reduced NAS (**Supplementary Figure 1**).

**Figure 3:**
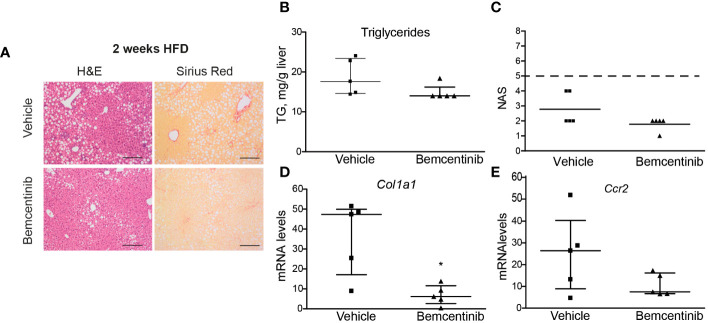
Bemcentinib administration prevents MASLD progression in 2-week HFD-fed mice. Liver sections of mice fed HFD for 2 weeks and treated with vehicle (*n* = 5) or bemcentinib 100 mg/kg (*n* = 5) for the last week, while on HFD, stained with H&E and Sirius Red **(A)**. Triglycerides in liver extracts **(B)** and NAS **(C)** were determined. RNA expression of Col1a1 and Ccr2 in liver samples **(D, E)**. Scale bar, 200 μm. H&E, hematoxylin and eosin; NAS, non-alcoholic fatty liver disease activity score. Mann–Whitney test; *p ≤ 0.05 vs. vehicle.

We then wanted to see if AXL inhibition could be used even earlier. We placed mice on HFD for 2 weeks and treated them with bemcentinib during the last week. One week of bemcentinib administration was enough to prevent MASLD progression in mice fed HFD for 2 weeks (**Figure 3A**). Of note, there was a detectable decrease in hepatic steatosis as observed in H&E-stained sections and in the reduction of liver triglycerides (**Figure 3B**), which is reflected in the NAS score (**Figure 3C**). Moreover, the levels of inflammatory and fibrotic genes such as *Ccr2* or *Col1a1* were also reduced by bemcentinib (**Figures 3D, E**) indicating that early AXL inhibition reduced MASLD development.

**Figure 4:**
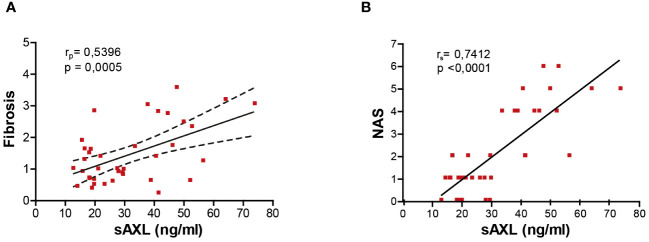
sAXL level is an early marker of liver fibrosis. Correlation of serum sAXL levels with liver fibrosis ImageJ quantification **(A)** and NAS **(B)**. The coefficient of determination (*r*) and statistical significance (*p*-value) were determined using Pearson’s coefficient **(A)** since both variables are continuous and Spearman’s coefficient **(B)** since one variable is continuous and the other is ordinal.

Several studies have proposed sAXL as a non-invasive fibrosis biomarker in hepatitis C virus (HCV), alcoholic steatohepatitis (ASH), and MASH patients (4, 7). Our previous results pointed to an increase of serum sAXL in MASLD patients before the onset of fibrosis (4). In accordance, the results of the different animal models of the present investigation show that serum sAXL correlates with fibrosis quantification (**Figure 4A**), but remarkably also with NAS (**Figure 4B**). Hence, sAXL is involved in the MASLD–MASH transition and its assessment in serum samples could represent an early marker of the disease, even before histological detection. Moreover, early treatment with bemcentinib prevented experimental MASLD/MASH progression, indicating that AXL inhibition is a possible strategy to be tested in clinical trials.

### 3.3 Immune gene expression analysis

**Figure 5:**
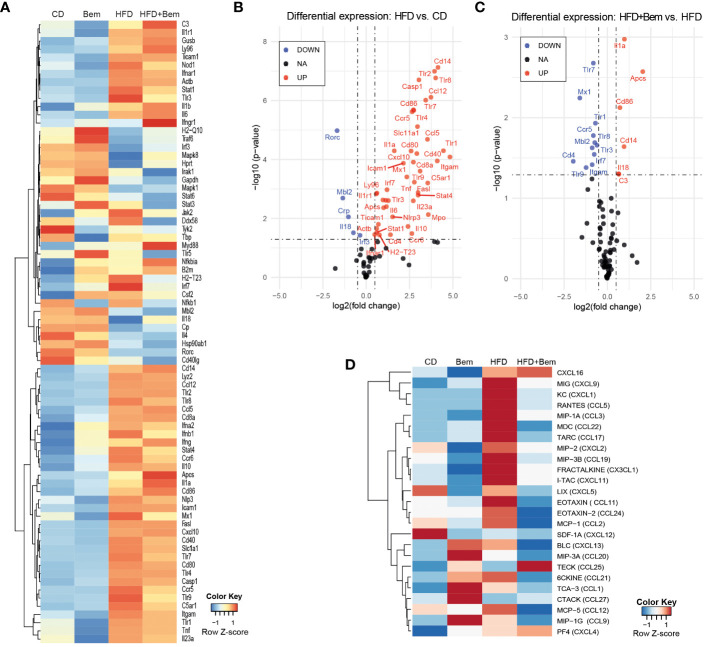
Changes in immune response mediators induced by bemcentinib. Heatmap of row-normalized transcriptomic changes (row *Z*-score) related to the innate and adaptive immune response in liver samples of mice fed with HFD or chow diet (8 weeks) with the addition of bemcentinib or vehicle gavages for the last 2 weeks while continuing on HFD **(A)**. Significantly upregulated genes are shown in red, while downregulated genes are in blue in the experimental MASH **(B)** and following bemcentinib treatment **(C)**. Cytokines and chemokines in mice sera were determined by a glass slide-based protein array, and relative levels were plotted in a heatmap (row *Z*-score) **(D)**. *n* = 2–5/group.

To further study the mechanism by which bemcentinib reduces liver injury, as shown above, we subjected mice to HFD or chow diet for 8 weeks and treated them with bemcentinib (100 mg/kg) or vehicle for the last 2 weeks and analyzed whether bemcentinib had measurable effects on immune-related transcripts. We analyzed changes in the innate and adaptive immune responses using a validated PrimePCR panel, and the resulting heatmap, row *Z*-score (**Figure 5A**), displays how treatment affects the overall gene expression. To look for statistical significance in these genes, volcano plots were used. As shown in **Figure 5B**, a multitude of inflammation-related genes are significantly upregulated in the liver of HFD-fed mice. Interestingly we also found downregulation of *Mbl2*, *Crp*, *Rorc*, and *Il18*. According to the Human Protein Atlas (22), in the liver, *Mbl2*, *Crp*, and *Rorc* were mainly expressed in hepatocytes, except for *Il18*, which was mainly expressed in Kupffer cells. Both *Mbl2* and *Crp* were tightly linked to the complement cascade through the lectin pathway and the classical pathway, respectively. The lack of *Mbl2* has previously been shown to exacerbate liver injury (23), while *Il18* has been shown to protect hepatocytes from apoptosis (24).

We next investigated the effect of bemcentinib (100 mg/kg vs. 0 mg/kg) in the HFD-fed mice. Despite the important hepatoprotection accompanied by bemcentinib treatment, only a few of the immune-related genes altered by HFD were significantly affected by the treatment, as shown in the volcano plot (**Figure 5C**). Focusing on these changes, transcription of Toll-like receptors (TLRs), which is frequently increased during chronic liver disease and in MASLD particularly (25), was markedly reduced after bemcentinib treatment. AXL inhibition, in addition to a marked reduction in the endosome-associated *Tlr7*, *Tlr8*, and *Tlr9*, strongly decreased the levels of *Tlr1* expression which has been positively associated with the bacterial translocation in MASH patients and proposed as a therapeutic target (26, 27). In addition, the levels of *Ccr5*, a cytokine receptor targeted in MASH therapy (28), were potently decreased by bemcentinib administration.

On the other side, a strong increase in the expression of *Amyloid P component, serum* (*Apcs*, *Ptx2*, *Sap*) was observed in bemcentinib-treated mice fed HFD (**Figure 5C**). *Apcs*, which is involved in binding microbes and activates the classical complement pathway (29–32), is reported to decline during MASLD progression (33), and its experimental administration effectively protects the steatotic liver (34). Of note, we also detected *Apcs* upregulation after bemcentinib administration in HFD mice after 2 and 4 weeks of feeding (unpublished results).

To verify if these changes in the immune system induced both by MASH and bemcentinib could be detected in the samples from these animals, we analyzed a serum cytokine protein array from all mice groups (vehicle *n* = 4, Bem *n* = 2, HFD *n* = 5, HFD+Bem *n* = 5). Heatmap, row *Z*-score, and representation of the arrays revealed that important changes in HFD-fed animals were partially abrogated by AXL inhibition (**Figure 5D**). Quantification of the arrays identified CXCL9, CXCL1, and CCL5 as cytokines highly elevated in MASH models that were diminished by bemcentinib. Of note, CCR5, whose transcription was reduced by AXL inhibition (**Figure 5C**), binds chemokines such as CCL5 or CCL2 and facilitates the recruitment of inflammatory cells to the site of inflammation. CCL3 followed an analogous pattern of MASH activation and bemcentinib reduction, the same as CCL2, which binds CCR2. Thus, AXL inhibition reduced CCR2 and CCR5 signaling similarly as the CCR2/5 antagonist cenicriviroc which is in advanced clinical trials for MASH (28). Other relevant cytokine alterations detected after bemcentinib administration that may be of interest are on CCL17 and CCL22 levels, principally produced by dendritic cells. CCL17 and CCL22 and their common receptor CCR4 seem to be associated with the promotion of inflammation in liver chronic diseases, Treg recruitment, and HCC promotion (35–37), and its repression could be another positive action of bemcentinib administration. In contrast, other chemokines such as CXCL16, clearly augmented in HFD-fed mice, were unaffected by AXL inhibition.

### 3.4 Bemcentinib administration at a wide range of doses reduces liver fibrosis and inflammation in a dietary MASH model

**Figure 6:**
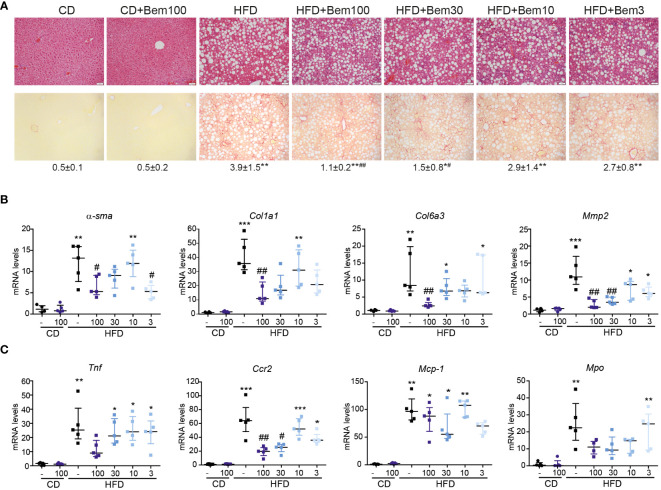
Dose–response effect of bemcentinib (Bem) in liver fibrosis in HFD-fed mice. Representative images of liver sections after H&E and Sirius Red staining from mice fed for 8 weeks with control diet (CD) and HFD diet that received vehicle or Bem for the last 2 weeks, while continuing on HFD, at different concentrations (100, 30, 10, and 3 mg/kg), *n* = 5 mice/group. Sirius Red quantifications are shown under representative pictures (mean ± SEM) **(A)**. Fibrogenic **(B)** and inflammatory **(C)** gene expression in the liver (fold change vs. CD). Kruskal–Wallis test; *p < 0.05, **p ≤ 0.01, and ***p < 0.001 vs. CD; #*p* < 0.05 and ##*p* ≤ 0.01vs. HFD; *n* = 5/group.

Above, we showed that bemcentinib acts on genes related to inflammation and alters cytokine production. To further study how bemcentinib ameliorated MASH development, we fed mice chow or HFD for 8 weeks and treated them with vehicle or bemcentinib (3, 10, 30, or 100 mg/kg) for the last 2 weeks. Using H&E and Sirius Red, we confirmed the development of steatosis and fibrosis with Sirius Red staining. Bemcentinib, particularly at 100 mg/kg and 30 mg/kg dosages, reproduced the positive results previously reported (4). A consistent reduction in collagen content, visualized in Sirius Red slides in bemcentinib doses from 100 to 3 mg/kg (**Figure 6A**), was accompanied by a significant transcriptional decrease in profibrotic (**Figure 6B**) and inflammation-related genes (**Figure 6C**), consistent with the beneficial effect of AXL inhibition during the development of experimental MASH (4). Although the 100- and 30-mg/kg dosages were most effective, dosages of 10 or 3 mg/kg also demonstrated beneficial results.

In relation to this, since the inhibitory effect of bemcentinib on *Mmp2* mRNA expression was quite remarkable at all the doses tested, we next analyzed the expression of other genes related to extracellular matrix reorganization, such as fibrosis-related metalloproteinases (*Mmp2*, *Mmp3*, or *Mmp13*) and tissue inhibitor metalloproteinases (*Timp1* or *Timp2*). As seen in **Supplementary Figure 2**, these genes were strongly upregulated by HFD intake, and bemcentinib markedly decreased its expression. On the other hand, repair-related metalloproteinases such as *Mmp9* and *Mmp8* (38–40) increased in HFD-fed mice and were not significantly affected by AXL inhibition (**Supplementary Figure 2**). Given that MMPs are differentially expressed depending on the cell type and their activity is strongly regulated by proteolytic activation and interaction with TIMPs (41), additional studies are needed to substantiate the impact of bemcentinib action on MMP/TIMP activity *in vivo*.

### 3.5 CyTOF captured the loss of Kupffer cells and increased immune cell infiltration during MASH development

**Figure 7:**
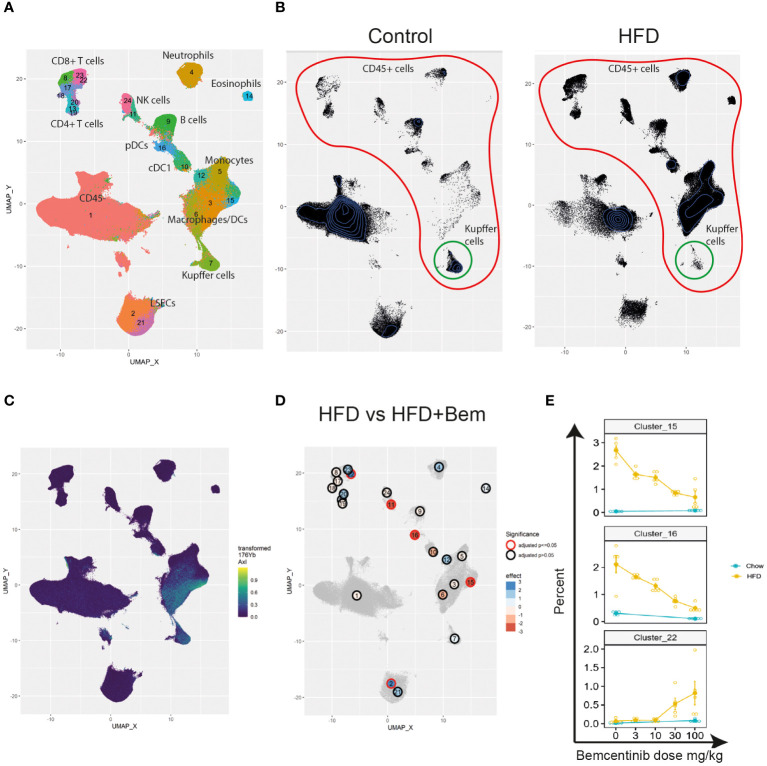
Single-cell analysis from dissociated liver reveals changes in immune cell populations induced by bemcentinib in experimental MASH. Clusters and cell types identified in the livers using CyTOF **(A)**. Comparison of UMAP density, immune cells (red), and Kupffer cells (green) in vehicle-treated mice fed control chow or HFD chow **(B)**. UMAP colored by AXL signal intensity **(C)** with cluster labels colored by the effect of bemcentinib treatment in HFD-fed mice (blue: higher in bemcentinib-treated, red: lower in bemcentinib-treated). The red circle indicates adjusted *p*-value <0.05 **(D)**. The percentages (mean ± SE) of the total selected clusters make up each sample across different dosages and diets **(E)**.

**Figure 8:**
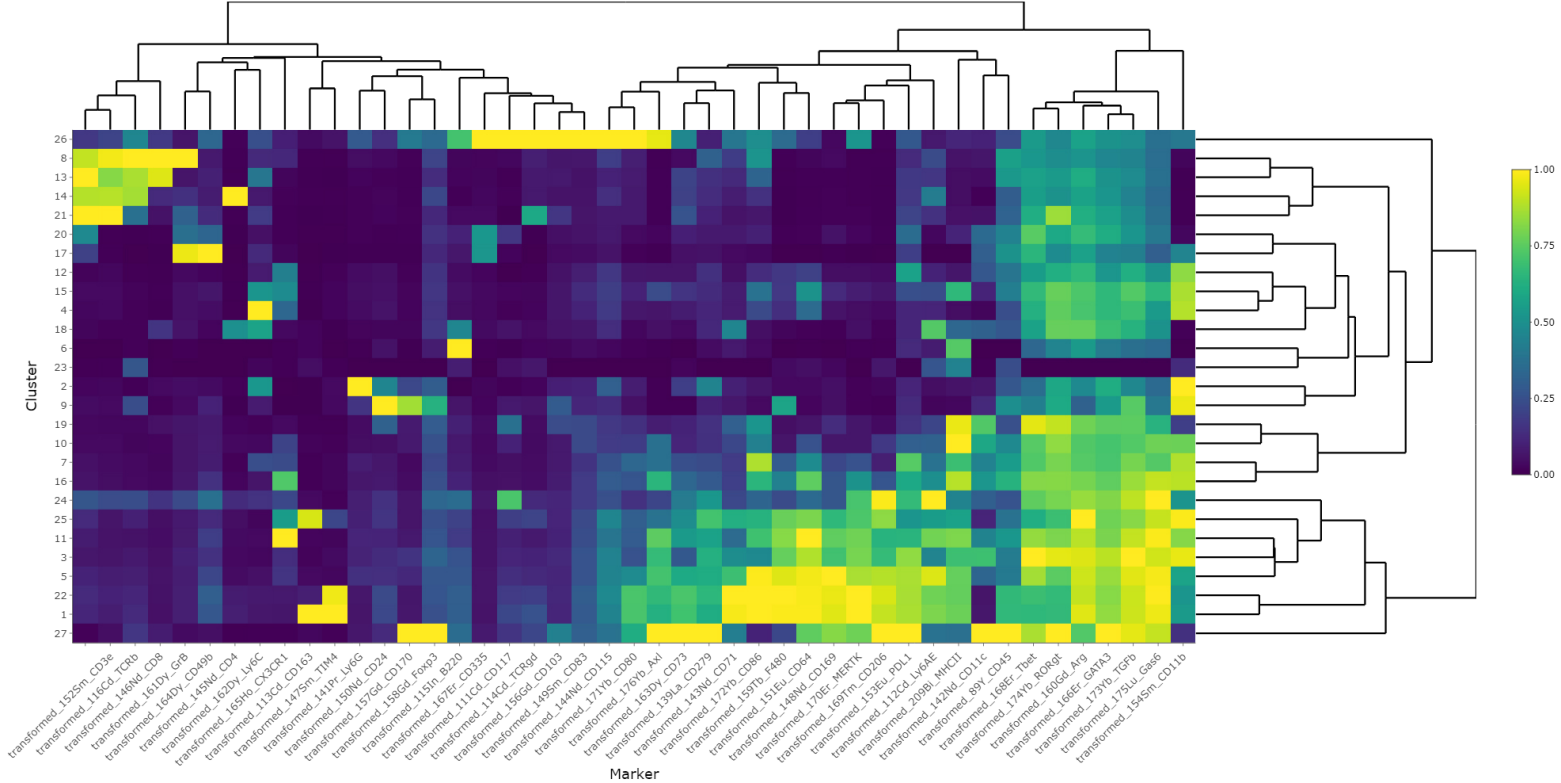
Heatmap of scaled marker expression of each cluster. The mean asinh-transformed signal intensity was calculated for each cluster. The means of each channel in each cluster were then column-scaled from 0 to 1. A higher value indicates a higher mean expression in the given cluster compared with other clusters.

Finally, to study the effects of bemcentinib on immune cell types in the liver, we established a 40-marker CyTOF antibody panel to be used on single cells from dissociated livers (**Supplementary Table 1**). We applied the panel on livers from mice fed chow or HFD for 8 weeks and treated with bemcentinib or vehicle for the last 2 weeks. After using PARC (20) to cluster the cells based on the staining of the antibody markers, we manually curated the clusters into 23 different populations based on marker expression (see **Figures 7A**, **8**).

We first aimed to distinguish untreated mice fed chow from untreated mice fed the HFD diet. By using UMAP (42) to visualize the high-dimensional data, we were able to capture changes in the liver cell type landscape imposed by the HFD in untreated mice (see **Figure 7B**). We found an increase in the transformed abundance of several types of immune cells, with a prominent increase in myeloid cells. Some immune cells did, however, demonstrate reduced transformed abundance, such as B cells (cluster 9), CD49b^high^GzmB^+^ NK cells (cluster 24), Kupffer cells (cluster 7), FOXP3^−^CD4^+^ T cells (cluster 13), and CD4^+^CD8^+^ T cells (cluster 20) (**Supplementary Figure 3**). HFD also had a significant effect on both CD117^low^CD73^low^ periportal and CD117^high^CD73^high^ pericentral endothelial cells which were reduced, compared with the livers of chow-fed mice.

We next identified AXL-expressing cells that are the primary targets of the treatment. We found that AXL was expressed in CD163^+^Tim4^+^ Kupffer cells (cluster 7), CD163^−^Tim4^−^CD11b^+^CD64^+^ monocyte-derived macrophages (clusters 3 and 6), and AXL^+^CD11c^+^CD11b^+^ monocyte-derived dendritic cells (cluster 15) (**Figure 7C**). MerTK, another member of the TAM receptor family along with AXL, was also expressed by CD163^−^Tim4^−^CD11b^+^CD64^+^ monocyte-derived macrophages (clusters 3 and 6) and Kupffer cells (cluster 7), similar to AXL, but in contrast to AXL, MerTK was expressed in endothelial cells (clusters 2 and 21), but not in AXL^+^CD11c^+^CD11b^+^ monocyte-derived dendritic cells (cluster 15) (**Supplementary Figure 4A**). Treatment with the specific AXL inhibitor bemcentinib, which has demonstrated a more than 50-fold selectivity to AXL than to the other TAM receptors (MerTK and TYRO3) (43), did not affect the MerTK cell expression pattern in the liver (**Supplementary Figure 4B**), confirming that the effects observed were primarily due to AXL inhibition. However, we cannot rule out that inhibition of AXL signaling affected GAS6 bioavailability and, consequently, MerTK-dependent signaling.

We next evaluated the differences between bemcentinib treatment (0 mg/kg vs. 100 mg/kg) in HFD-fed mice. Since the sum of the percentages of each cluster in every sample is constrained to 100%, the assumptions of classical statistical tests are not met; hence, special methods for compositional data must be applied. We therefore used ALDEx2 (21, 44, 45) to test for differences between these groups (see **Supplementary Table 3**). We found, **Figure 7D** that 100 mg/kg of bemcentinib significantly decreased the transformed abundance of CD49b^low^CD335^+^GzmB^−^ NK cells (cluster 11), Axl^+^CD11c^+^CD11b^+^ dendritic cells (cluster 15), and plasmacytoid dendritic cells (pDCs) (cluster 16) and increased CD73^low^CD117^low^ LSECs (cluster 2) reported to be periportal LSECs (46) and led to the emergence of a CX3CR1^+^CD8^+^GzmB^+^ T-cell subset (cluster 22) reported to be effector memory T cells (Tem) (47). These clusters also demonstrated the dose-dependent effects of bemcentinib treatment (**Figure 7E**). Axl^+^CD11c^+^CD11b^+^ DCs (cluster 15) and pDCs (cluster 16) both demonstrated a dose-dependent reduction with increasing dose of bemcentinib, while the Tem cells (cluster 22) had an opposite effect of increasing only in high-dose bemcentinib treatments. Periportal LSECs (cluster 2) and CD49b^low^CD335^+^GzmB^−^ NK cells (cluster 11) showed similar trends of dose-dependent increase and decrease, respectively (**Supplementary Figure 3**).

We also studied the effects of bemcentinib treatment in chow-fed mice. Specifically, we found a reduction in CD45^−^ cells (cluster 1), neutrophils (cluster 4), cDC1s (cluster 10), GzmB^−^ NK cells (cluster 11), and pDCs (cluster 16) and an increase of periportal endothelial cells (clusters 2), CD4^+^CD8^+^ T cells (cluster 20), and GzmB^+^CD8^+^ T cells (cluster 23) (see **Supplementary Table 4**). Of these, the largest effects were the reduction of pDCs (cluster 16) and the increase of GzmB^+^CD8^+^ T cells (cluster 23) and periportal LSECs (cluster 2). When comparing the clusters that were significantly affected by bemcentinib in either diet, we found that only neutrophils displayed opposing effects with a decrease in chow-fed mice and an increase in HFD-fed mice.

**Figure 9:**
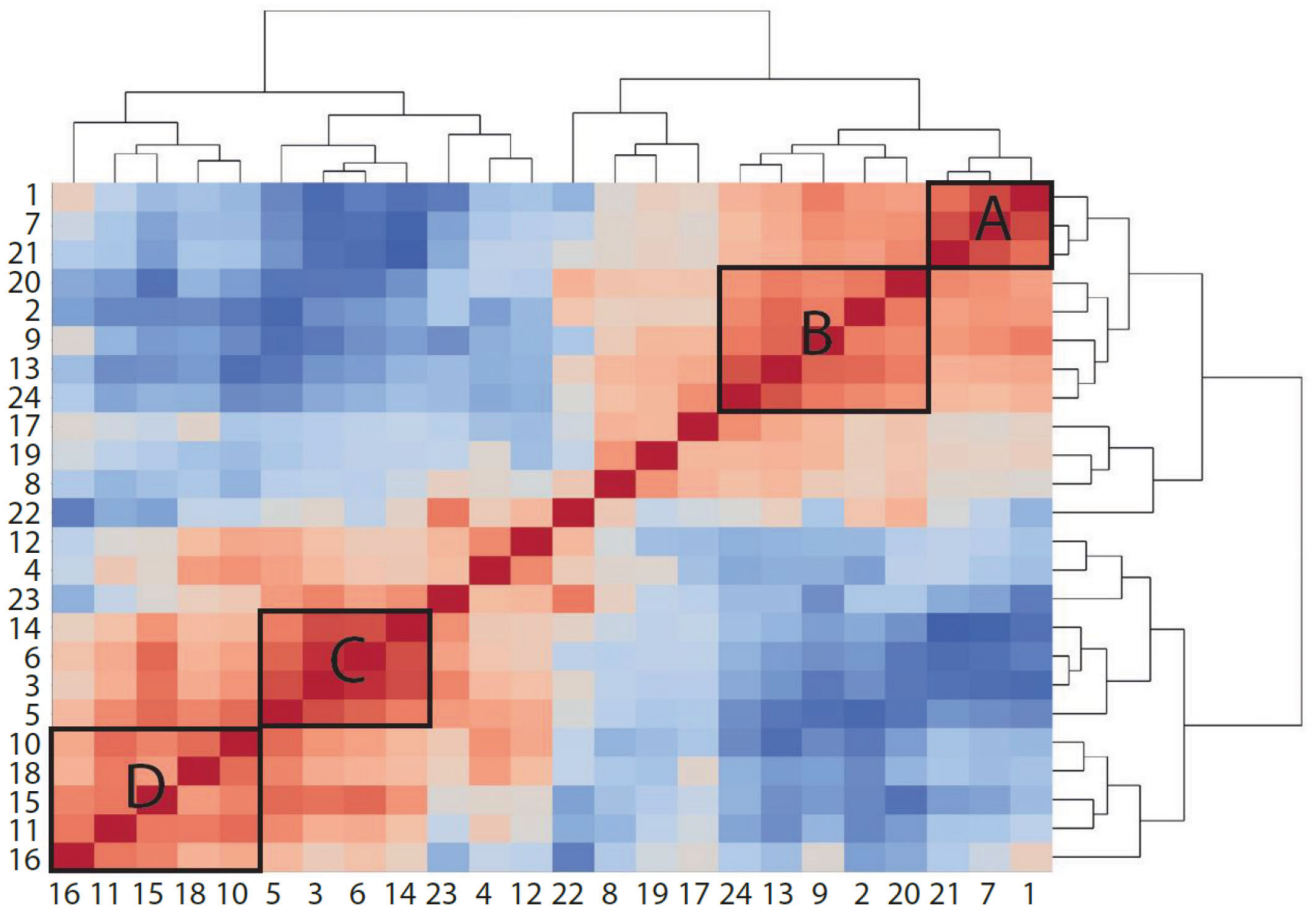
Proportionality between clusters suggests cell types that increase or decrease together. Heatmap colored by proportionality (rho) between clusters. Red is positive proportionality and blue is negative. A, B, C, and D label different groups of clusters that are proportional to each other. A and B are generally reduced in mice fed HFD, while C and D are increased.

When comparing the effects of bemcentinib on immune cell populations, we observed that several clusters responded in a similar manner. To study whether any cell types correlated with each other across samples, we measured proportionality between clusters using rho (48) (**Figure 9**). The two highest proportionalities were found between macrophages (clusters 3 and 6) (rho = 0.9438) and between Kupffer cells (cluster 7) and CD45^−^ cells (cluster 1) (rho = 0.8722). The two lowest proportionalities were found between eosinophils (cluster 14) and central LSECs (cluster 21) (rho = −0.9442) and between eosinophils and Kupffer cells (cluster 7) (rho = −0.8978). Some cell types also formed groups of high proportionalities, such as monocytes, macrophages, and eosinophils (group C, clusters 3, 5, 6, and 14) and Kupffer cells, pericentral CD117^high^ LSECs, and CD45^−^ cells (group A, clusters 1, 7, and 21). As expected, these two groups displayed a high negative proportionality between each other, as Kupffer cells, central LSECs, and CD45^−^ cells are reduced upon the infiltration of eosinophils and monocytes, which differentiate into macrophages. pDCs (cluster 16) displayed a highly negative proportionality with CX3CR1^+^CD8^+^ T cells (cluster 22) and Gzmb^+^CD8^+^ T cells (cluster 23).

## 4 Discussion

The present study provides valuable insights into the role of the GAS6/TAM pathway in the progression of MASLD and MASH. The findings highlight the relevance of the cleaved extracellular domain of the AXL receptor, sAXL, as an early biomarker of MASLD/MASH, before the onset of histological fibrosis, and support the potential therapeutic use of AXL inhibition in preventing disease progression.

### 4.1 sAXL

We observed a marked increase of serum sAXL in mice fed WD, which exhibited fatty livers with significant fibrosis and inflammation, in line with our previous results with a high-fat, choline-deficient, and methionine-restricted diet (HFD). In contrast, the HFF diet model, which displayed liver steatosis without concurrent development of fibrosis or significant inflammation, did not show an increase in sAXL levels. This observation supports that the presence of steatosis alone is not sufficient to trigger the activation of the GAS6/TAM pathway and the release of sAXL, revealed to be an early biomarker of MASLD/MASH progression. Indeed, inflammatory mediators induce AXL expression in macrophages, dendritic cells, and microglia (49–51), while the same treatments inhibit MerTK expression. It is possible that inflammatory stimuli are priming the GAS6/TAM system for an adaptative response. Previous studies have determined sAXL in the plasma/serum from patients of different liver diseases, showing a concordant behavior where sAXL levels increase proportionally to the severity of the disease and are indicative of worse clinical outcomes (4, 7, 52–55). Our data using diverse animal models support this view and indicate that inflammation and fibrosis are critical factors in this increase. Using mass cytometry to study the livers of HFD-fed mice, we found that AXL expression is dominated by monocyte-derived macrophages, implicating them as a source of sAXL. In a broader perspective and in concordance with our results, monocyte-derived macrophages have been shown to appear concurrent with the loss of tissue-resident macrophages in several tissues including the liver, brain, lungs, and heart, during events of sustained tissue damage (56, 57). Damage in these same tissues (cirrhosis, Alzheimer’s disease, pneumonia, and heart failure) has also been directly linked to increased levels of sAXL (53, 58–60). This suggests that an elevated sAXL level serves as an indicator of ongoing tissue damage that initially depletes tissue-resident macrophages, leading to their replacement by monocyte-derived macrophages, which regrettably are unable to resolve the sustained tissue damage and resultant inflammation.

### 4.2 CyTOF

A notable constraint in CyTOF experiments stems from the compositional nature of the data generated. Specifically, the loss of a particular cell type, such as through necrosis, is indistinguishable from the emergence of another cell type. Both scenarios manifest as a diminished proportion of one or more cell types and a corresponding increase in others, thereby posing a challenge to accurately interpret the cellular dynamics at play. Nonetheless, our results do recapitulate previously demonstrated features of MASH, such as loss of Kupffer cells and infiltration of monocytes (56, 61).

Our study demonstrates the potential therapeutic efficacy of AXL inhibition using the specific AXL inhibitor bemcentinib and provides clues for its therapeutic action. We found that bemcentinib treatment in HFD-fed mice showed dose-dependent effects on reducing the transformed abundance of pDCs, CD335^+^GzmB^−^ NK cells, and Axl^+^CD11c^+^CD11b^+^DCs and increasing the transformed abundance of CD117^low^ periportal LSECs and CX3CR1^+^CD8^+^GzmB^+^ Tem. The decrease in DCs is consistent with the reduction in CCL17 and CCL22 reported in **Figure 5D** in the serum. In chow-fed mice, the strongest effects of bemcentinib were the observed loss of pDCs and the increase of portal LSECs, which is consistent with the observations in HFD-fed mice. Neither of these populations expressed AXL; however, LSECs abundantly expressed MerTK, which shares the AXL’s ligand GAS6. We have previously shown that bemcentinib increases the levels of serum GAS6 in mice (4), and we speculate that bemcentinib-driven increases in serum GAS6 could result in increased MerTK signaling, for example, in endothelial cells. LSECs have previously been shown to play an important role in the progression of MASH. Indeed, loss of LSEC fenestrations is an early sign of MASLD, and they are suggested to act as “gatekeepers” in the development of the disease (62). LSECs are further directly implicated in the zonation of immune cells, as they have been shown to determine Kupffer cell positioning through the regulation of the extracellular matrix and the glycocalyx (63). Our results demonstrate that the transformed abundance of LSECs is significantly reduced by HFD and that bemcentinib partially restores periportal LSECs. Peiseler and colleagues have demonstrated that liver fibrosis is associated with sinusoidal constriction and increased collateral vessel formation, suggesting a diminished presence of LSECs—a finding that aligns with our observations (61). Furthermore, bemcentinib had a positive effect on increasing the transformed abundance of Kupffer cells, albeit not statistically significant. We also found a particularly high proportionality between LSECs and Kupffer cells; however, in contrast to the reported preferential periportal positioning of Kupffer cells, we found that CD73^high^CD117^high^ pericentral LSECs had higher proportionality with Kupffer cells than portal LSECs. It is possible that early MASH alters periportal LSECs or induces injury in the pericentral hepatocytes which results in pericentral relocation of Kupffer cells.

The loss of pDCs observed in the CyTOF experiments is also supported by the observed reduced transcription of *Cd4*, *Tlr7*, *Tlr9*, and *Irf7*, all of which are proteins known to be expressed in pDCs (64). However, these proteins are somewhat ubiquitously expressed in mice. For example, *Tlr7* has been shown to be expressed in both monocyte-derived cells and DCs (65). In the context of MASH, pDCs have been largely understudied; however, they are known for their abilities to recognize nucleic acids and to secrete excessive amounts of type 1 interferons and have been shown to be increased during MASH in both mice and humans (66). In a mouse model of diet-induced obesity and type 2 diabetes, it was demonstrated that the abundance of pDCs was increased in the liver of diseased mice, and their depletion resulted in improvement (67). Furthermore, while not directly measuring pDCs, TLR9 has also been shown to drive MASH by recognizing hepatocyte-derived mitochondrial DNA, possibly released during cell death or high stress, while *Tlr9* knockout mice are protected (27, 68).

The reduction of pDCs upon bemcentinib treatment could, however, indicate increased IFN-1 secretion, as they have been shown to undergo apoptosis when exposed to high amounts of IFN-1 (69). The loss of pDCs coincided with an increase of Gzm^+^CD8^+^ T cells (clusters 22 and 23) as demonstrated by their negative proportionality. Previously, tissue-resident memory CD8^+^ T cells have been implicated in driving the resolution of fibrosis by killing stellate cells (70). That paper did not report on CX3CR1 expression; however, others have characterized CX3CR1^+^Gzmb^+^CD8^+^ T cells (cluster 22) as effector memory T cells (47). CX3CR1^+^Gzmb^+^CD8^+^ T cells were found at a substantially higher abundance in high-dose bemcentinib-treated MASH livers, which coincided with low amounts of pDCs. Our findings indicate that bemcentinib both augments the population of effector memory T cells and diminishes the abundance of pDCs. While a causal relationship has not been established, it is plausible that the observed increase in effector memory T cells may result from bemcentinib’s reduction of pDCs.

### 4.3 mRNA

Overall, this increased expression of *APCS* could reflect the reduction of proinflammatory signaling in the liver, indicating the resolution of tissue damage and regeneration of the liver parenchyma. Therefore, AXL inhibition seems to be affecting the expression of *TLRs* and *CCR5* in a way that shifts the immune response toward a more anti-inflammatory and tissue-remodeling phenotype, which could contribute to the observed increase in CX3CR1 and pentraxin-2 expression.

The transcriptomic analysis further supports the involvement of AXL inhibition in modulating the immune response in MASLD/MASH. The results indicate a reduction in Toll-like receptors (TLRs) and CCR5 expression upon AXL inhibition. TLRs play a crucial role in recognizing pathogen-associated molecular patterns triggering innate immune responses and directly regulate the expression of TAMs in immune cells (49). Their dysregulation has been implicated in the progression of liver disease. CCR5, a chemokine receptor, is involved in the recruitment and activation of inflammatory cells, including macrophages and T cells, in various liver diseases. The downregulation of TLRs and CCR5 observed upon AXL inhibition suggests a potential mechanism by which AXL antagonism may attenuate the inflammatory response in MASLD/MASH. These findings provide further support for the immunomodulatory effects of AXL inhibition in the context of liver disease (7, 8, 15, 16, 71).

Regarding this, we should consider that bemcentinib administration increases the levels of sAXL and GAS6 in the serum (4). Due to the ability of GAS6 to bind phosphatidylserine (PS) and facilitate the clearance of apoptotic cells, this mechanism may contribute to diminishing TLR activation. GAS6 acts as a bridging molecule by binding to PS on apoptotic cells and engaging with receptors on phagocytes, such as TAM receptors (including AXL). GAS6-mediated efferocytosis contributes to protection in different contexts. In autoimmune diseases, such as systemic lupus erythematosus (SLE), where impaired clearance of apoptotic cells leads to the release of self-antigens, this triggers an autoimmune response that GAS6 dampens. In inflammatory lung diseases, GAS6 promotes efferocytosis by alveolar macrophages in the lungs, preventing excessive inflammation and tissue damage. In neurodegenerative diseases, characterized by the accumulation of apoptotic cells and debris in the brain, GAS6 enhances the clearance of apoptotic neurons by microglial cells and reduces neuroinflammation. In the context of MASH, by efficiently removing apoptotic cells, GAS6 could prevent the release of damage-associated molecular patterns (DAMPs) that would otherwise activate TLRs and perpetuate liver inflammation. Of note, the restorative CX3CR1^+^ MoMF subset increased by bemcentinib showed a notable expression of MerTK which may contribute to the resolution of liver damage through efferocytosis and the derived anti-inflammatory signaling (72).

Our study focused primarily on the effects of AXL inhibition on liver steatosis, inflammation, and fibrosis. However, MASLD/MASH is a complex disease involving multiple organ systems, including adipose tissue, the gut microbiota, and systemic inflammation. The role of the microbiota in MASH is an area of active research, and emerging evidence suggests that alterations in the gut microbiota composition and function may contribute to the development and progression of MASH. While the direct effect of AXL inhibition on the microbiota in the context of MASH is not well-studied, there is some evidence to suggest that AXL signaling may indirectly influence the gut microbiota and its impact on MASH. In this sense, AXL signaling has been implicated in maintaining intestinal barrier integrity. Translocation of bacterial products, such as lipopolysaccharides (LPS), from the gut lumen into the systemic circulation, contributes to the pathogenesis of MASH. Bemcentinib may influence the interaction between the microbiota and MASH development by reducing the translocation of proinflammatory molecules from the gut that would impact the inflammatory response and indirectly by changing immune cell subsets. Similarly, AXL inhibition could alter bile acid metabolism and/or influence gut microbiota-mediated bile acid modifications, thereby impacting MASH pathogenesis.

In conclusion, this study sheds light on the role of the GAS6/TAM pathway and sAXL in the pathogenesis of MASLD/MASH. The findings support the potential of sAXL as an early biomarker for MASLD/MASH and highlight the therapeutic efficacy of AXL inhibition in attenuating liver steatosis, inflammation, and fibrosis. Furthermore, the study provides insights into the immunomodulatory effects of AXL inhibition and the potential modulation of immune cell populations in MASLD/MASH. While further research is needed to elucidate the underlying mechanisms and explore the translational potential, these findings contribute to our current knowledge and offer promising avenues for the development of targeted therapies for MASLD/MASH.

## Data availability statement

The original contributions presented in the study are included in the article/**Supplementary Material**. Further inquiries can be directed to the corresponding authors.

## Ethics statement

The animal study was approved by Comité d’ètica d’experimetació Animal de la Universitat de Barcelona (CEEA-UB). The study was conducted in accordance with the local legislation and institutional requirements.

## Author contributions

SG: Data curation, Formal analysis, Investigation, Methodology, Software, Validation, Visualization, Writing – original draft, Writing – review & editing. AT: Data curation, Formal analysis, Investigation, Methodology, Validation, Visualization, Writing – review & editing. LB: Data curation, Investigation, Supervision, Visualization, Writing – review & editing. MR: Investigation, Methodology, Visualization, Writing – review & editing. MB: Investigation, Visualization, Writing – review & editing. GG: Funding acquisition, Investigation, Methodology, Resources, Visualization, Writing – review & editing. LH: Funding acquisition, Investigation, Methodology, Resources, Visualization, Writing – review & editing. AJ: Funding acquisition, Methodology, Resources, Visualization, Writing – review & editing. PG: Formal analysis, Funding acquisition, Investigation, Methodology, Resources, Validation, Visualization, Writing – review & editing. JL: Supervision, Validation, Visualization, Writing – original draft, Writing – review & editing, Conceptualization, Data curation, Formal analysis, Funding acquisition, Investigation, Methodology, Project administration, Resources. AM: Conceptualization, Data curation, Formal analysis, Funding acquisition, Investigation, Methodology, Project administration, Resources, Supervision, Validation, Visualization, Writing – original draft, Writing – review & editing. MM: Conceptualization, Data curation, Formal analysis, Funding acquisition, Investigation, Methodology, Project administration, Resources, Supervision, Validation, Visualization, Writing – original draft, Writing – review & editing.

## References

[1] Rinella ME. Nonalcoholic fatty liver disease: a systematic review. JAMA. (2015) 313:2263–73. doi: 10.1001/jama.2015.5370 26057287

[2] Younossi ZM Koenig AB Abdelatif D Fazel Y Henry L Wymer M. Global epidemiology of nonalcoholic fatty liver disease-Meta-analytic assessment of prevalence, incidence, and outcomes. Hepatol Baltim. Md. (2016) 64:73–84. doi: 10.1002/hep.28431 26707365

[3] Schuppan D Surabattula R Wang XY. Determinants of fibrosis progression and regression in NASH. J Hepatol. (2018) 68:238–50. doi: 10.1016/j.jhep.2017.11.012 29154966

[4] Tutusaus A de Gregorio E Cucarull B Cristóbal H Aresté C Graupera I. A functional role of GAS6/TAM in nonalcoholic steatohepatitis progression implicates AXL as therapeutic target. Cell. Mol. Gastroenterol. Hepatol. (2020) 9:349–68. doi: 10.1016/j.jcmgh.2019.10.010 PMC7013198 31689560

[5] Cai B Dongiovanni P Corey KE Wang X Shmarakov IO Zheng Z. Macrophage merTK promotes liver fibrosis in nonalcoholic steatohepatitis. Cell Metab. (2020) 31:406–421.e7. doi: 10.1016/j.cmet.2019.11.013 31839486 PMC7004886

[6] Pastore M Caligiuri A Raggi C Navari N Piombanti B Di Maira G. Macrophage MerTK promotes profibrogenic cross-talk with hepatic stellate cells via soluble mediators. JHEP Rep Innov Hepatol. (2022) 4:100444. doi: 10.1016/j.jhepr.2022.100444 PMC8891698 35252828

[7] Bárcena C Stefanovic M Tutusaus A Joannas L Menéndez A García-Ruiz C. Gas6/Axl pathway is activated in chronic liver disease and its targeting reduces fibrosis via hepatic stellate cell inactivation. J Hepatol. (2015) 63:670–8. doi: 10.1016/j.jhep.2015.04.013 PMC4543529 25908269

[8] Zagórska A Través PG Jiménez-García L Strickland JD Oh J Tapia FJ. Differential regulation of hepatic physiology and injury by the TAM receptors Axl and Mer. Life Sci Alliance. (2020) 3:e202000694. doi: 10.26508/lsa.202000694 32571802 PMC7335405

[9] Geng K Kumar S Kimani SG Kholodovych V Kasikara C Mizuno K. Requirement of gamma-carboxyglutamic acid modification and phosphatidylserine binding for the activation of tyro3, axl, and mertk receptors by growth arrest-specific 6. Front Immunol. (2017) 8:1521. doi: 10.3389/fimmu.2017.01521 29176978 PMC5686386

[10] Lemke G Rothlin CV. Immunobiology of the TAM receptors. Nat Rev Immunol. (2008) 8:327–36. doi: 10.1038/nri2303 PMC2856445 18421305

[11] Graham DK DeRyckere D Davies KD Earp HS. The TAM family: phosphatidylserine sensing receptor tyrosine kinases gone awry in cancer. Nat Rev Cancer. (2014) 14:769–85. doi: 10.1038/nrc3847 25568918

[12] Ludwig KF Du W Sorrelle NB Wnuk-Lipinska K Topalovski M Toombs JE. Small-molecule inhibition of axl targets tumor immune suppression and enhances chemotherapy in pancreatic cancer. Cancer Res. (2018) 78:246–55. doi: 10.1158/0008-5472.CAN-17-1973 PMC5754222 29180468

[13] Engelsen AST Lotsberg ML Abou Khouzam R Thiery J-P Lorens JB Chouaib S. Dissecting the role of AXL in cancer immune escape and resistance to immune checkpoint inhibition. Front Immunol. (2022) 13:869676. doi: 10.3389/fimmu.2022.869676 35572601 PMC9092944

[14] Couchie D Lafdil F Martin-Garcia N Laperche Y Zafrani ES Mavier P. Expression and role of Gas6 protein and of its receptor Axl in hepatic regeneration from oval cells in the rat. Gastroenterology. (2005) 129:1633–42. doi: 10.1053/j.gastro.2005.08.004 16285961

[15] Pop O-T Geng A Flint E Singanayagam A Ercan C Possamai L. AXL expression on homeostatic resident liver macrophages is reduced in cirrhosis following GAS6 production by hepatic stellate cells. Cell Mol Gastroenterol Hepatol. (2023), S2352-345X(23)00047-4. doi: 10.1016/j.jcmgh.2023.03.007. PMC10209017 37004869

[16] Tutusaus A Morales A García de Frutos P Marí M. GAS6/TAM axis as therapeutic target in liver diseases. Semin Liver Dis. (2024) 44:99–114. doi: 10.1055/a-2275-0408 38395061 PMC11027478

[17] Asgharpour A Cazanave SC Pacana T Seneshaw M Vincent R Banini BA. A diet-induced animal model of non-alcoholic fatty liver disease and hepatocellular cancer. J Hepatol. (2016) 65:579–88. doi: 10.1016/j.jhep.2016.05.005 PMC5012902 27261415

[18] Bedossa P Poitou C Veyrie N Bouillot JL Basdevant A Paradis V. Histopathological algorithm and scoring system for evaluation of liver lesions in morbidly obese patients. Hepatol Baltim. Md. (2012) 56:1751–9. doi: 10.1002/hep.25889 22707395

[19] Chevrier S Crowell HL Zanotelli VRT Engler S Robinson MD Bodenmiller B. Compensation of signal spillover in suspension and imaging mass cytometry. Cell Syst. (2018) 6:612–620.e5. doi: 10.1016/j.cels.2018.02.010 29605184 PMC5981006

[20] Stassen SV Siu DMD Lee KCM Ho JWK So HKH Tsia KK. PARC: ultrafast and accurate clustering of phenotypic data of millions of single cells. Bioinformatics. (2020) 36:2778–86. doi: 10.1093/bioinformatics/btaa042 PMC7203756 31971583

[21] Fernandes AD Macklaim JM Linn TG Reid G Gloor GB. ANOVA-like differential expression (ALDEx) analysis for mixed population RNA-seq. PloS One. (2013) 8:e67019. doi: 10.1371/journal.pone.0067019 23843979 PMC3699591

[22] Karlsson M Zhang C Méar L Zhong W Digre A Katona B. A single-cell type transcriptomics map of human tissues. Sci Adv. (2021) 7:eabh2169. doi: 10.1126/sciadv.abh2169 34321199 PMC8318366

[23] Zhou J Li J Yu Y Liu Y Li H Liu Y. Mannan-binding lectin deficiency exacerbates sterile liver injury in mice through enhancing hepatic neutrophil recruitment. J Leukoc. Biol. (2019) 105:177–86. doi: 10.1002/JLB.3A0718-251R 30351498

[24] Yamamoto N Murata K Yoneda K Fuke H Yamaguchi Y Ito K. Protective role of interleukin-18 against Fas-mediated liver injury. Int J Mol Med. (2008) 22:43–8. doi: 10.3892/ijmm 18575774

[25] Mencin A Kluwe J Schwabe RF. Toll-like receptors as targets in chronic liver diseases. Gut. (2009) 58:704–20. doi: 10.1136/gut.2008.156307 PMC2791673 19359436

[26] Baumann A Nier A Hernández-Arriaga A Brandt A Lorenzo Pisarello MJ Jin CJ. Toll-like receptor 1 as a possible target in non-alcoholic fatty liver disease. Sci Rep. (2021) 11:17815. doi: 10.1038/s41598-021-97346-9 34497333 PMC8426394

[27] Miura K Kodama Y Inokuchi S Schnabl B Aoyama T Ohnishi H. Toll-like receptor 9 promotes steatohepatitis by induction of interleukin-1beta in mice. Gastroenterology. (2010) 139:323–334.e7. doi: 10.1053/j.gastro.2010.03.052 20347818 PMC4631262

[28] Friedman SL Ratziu V Harrison SA Abdelmalek MF Aithal GP Caballeria J. A randomized, placebo-controlled trial of cenicriviroc for treatment of nonalcoholic steatohepatitis with fibrosis. Hepatol Baltim. Md. (2018) 67:1754–67. doi: 10.1002/hep.29477 PMC5947654 28833331

[29] Behrens NE Lipke PN Pilling D Gomer RH Klotz SA. Serum amyloid P component binds fungal surface amyloid and decreases human macrophage phagocytosis and secretion of inflammatory cytokines. mBio. (2019) 10:e00218–19. doi: 10.1128/mBio.00218-19 PMC6414697 30862745

[30] Doni A Parente R Laface I Magrini E Cunha C Colombo FS. Serum amyloid P component is an essential element of resistance against Aspergillus fumigatus. Nat Commun. (2021) 12:3739. doi: 10.1038/s41467-021-24021-y 34145258 PMC8213769

[31] An J-H Kurokawa K Jung D-J Kim M-J Kim C-H Fujimoto Y. Human SAP is a novel peptidoglycan recognition protein that induces complement-independent phagocytosis of Staphylococcus aureus. J Immunol Baltim. Md 1950. (2013) 191:3319–27. doi: 10.4049/jimmunol.1300940 PMC4277995 23966633

[32] Noursadeghi M Bickerstaff MC Gallimore JR Herbert J Cohen J Pepys MB. Role of serum amyloid P component in bacterial infection: protection of the host or protection of the pathogen. Proc Natl Acad Sci U. S. A. (2000) 97:14584–9. doi: 10.1073/pnas.97.26.14584 PMC18962 11121061

[33] Verna EC Patel J Bettencourt R Nguyen P Hernandez C Valasek MA. Novel association between serum pentraxin-2 levels and advanced fibrosis in well-characterised patients with non-alcoholic fatty liver disease. Aliment. Pharmacol Ther. (2015) 42:582–90. doi: 10.1111/apt.13292 PMC7979413 26119353

[34] Pilling D Cox N Thomson MA Karhadkar TR Gomer RH. Serum amyloid P and a dendritic cell-specific intercellular adhesion molecule-3-grabbing nonintegrin ligand inhibit high-fat diet-induced adipose tissue and liver inflammation and steatosis in mice. Am J Pathol. (2019) 189:2400–13. doi: 10.1016/j.ajpath.2019.08.005 PMC6902115 31539521

[35] Oo YH Weston CJ Lalor PF Curbishley SM Withers DR Reynolds GM. Distinct roles for CCR4 and CXCR3 in the recruitment and positioning of regulatory T cells in the inflamed human liver. J Immunol Baltim. Md 1950. (2010) 184:2886–98. doi: 10.4049/jimmunol.0901216 20164417

[36] Riezu-Boj J-I Larrea E Aldabe R Guembe L Casares N Galeano E. Hepatitis C virus induces the expression of CCL17 and CCL22 chemokines that attract regulatory T cells to the site of infection. J Hepatol. (2011) 54:422–31. doi: 10.1016/j.jhep.2010.07.014 21129807

[37] Cheng X Wu H Jin Z-J Ma D Yuen S Jing X-Q. Up-regulation of chemokine receptor CCR4 is associated with Human Hepatocellular Carcinoma Malignant behavior. Sci Rep. (2017) 7:12362. doi: 10.1038/s41598-017-10267-4 28959024 PMC5620046

[38] Siller-López F Sandoval A Salgado S Salazar A Bueno M Garcia J. Treatment with human metalloproteinase-8 gene delivery ameliorates experimental rat liver cirrhosis. Gastroenterology. (2004) 126:1122–1133; discussion 949. doi: 10.1053/j.gastro.2003.12.045 15057751

[39] Wen G Zhang C Chen Q Luong LA Mustafa A Ye S. A novel role of matrix metalloproteinase-8 in macrophage differentiation and polarization. J Biol Chem. (2015) 290:19158–72. doi: 10.1074/jbc.M114.634022 PMC4521038 26092731

[40] Feng M Ding J Wang M Zhang J Zhu X Guan W. Kupffer-derived matrix metalloproteinase-9 contributes to liver fibrosis resolution. Int J Biol Sci. (2018) 14:1033–40. doi: 10.7150/ijbs.25589 PMC6036732 29989076

[41] Yamamoto K Murphy G Troeberg L. Extracellular regulation of metalloproteinases. Matrix Biol J Int Soc Matrix Biol. (2015) 44–46:255–63. doi: 10.1016/j.matbio.2015.02.007 25701651

[42] McInnes L Healy J Melville J. UMAP: uniform manifold approximation and projection for dimension reduction. (2020). doi: 10.48550/arXiv.1802.03426

[43] Holland SJ Pan A Franci C Hu Y Chang B Li W. R428, a selective small molecule inhibitor of Axl kinase, blocks tumor spread and prolongs survival in models of metastatic breast cancer. Cancer Res. (2010) 70:1544–54. doi: 10.1158/0008-5472.CAN-09-2997 20145120

[44] Gloor GB Macklaim JM Fernandes AD. Displaying variation in large datasets: plotting a visual summary of effect sizes. J Comput Graph. Stat. (2016) 25:971–9. doi: 10.1080/10618600.2015.1131161

[45] Unifying the analysis of high-throughput sequencing datasets: characterizing RNA-seq, 16S rRNA gene sequencing and selective growth experiments by compositional data analysis. Microbiome. doi: 10.1186/2049-2618-2-15 PMC4030730 24910773

[46] Halpern KB Shenhav R Massalha H Toth B Egozi A Massasa EE. Paired-cell sequencing enables spatial gene expression mapping of liver endothelial cells. Nat Biotechnol. (2018) 36:962–70. doi: 10.1038/nbt.4231 PMC6546596 30222169

[47] Gerlach C Moseman EA Loughhead SM Alvarez D Zwijnenburg AJ Waanders L. The chemokine receptor CX3CR1 defines three antigen-experienced CD8 T cell subsets with distinct roles in immune surveillance and homeostasis. Immunity. (2016) 45:1270–84. doi: 10.1016/j.immuni.2016.10.018 PMC5177508 27939671

[48] Quinn TP Richardson MF Lovell D Crowley TM. propr: an R-package for identifying proportionally abundant features using compositional data analysis. Sci Rep. (2017) 7:16252. doi: 10.1038/s41598-017-16520-0 29176663 PMC5701231

[49] Zagórska A Través PG Lew ED Dransfield I Lemke G. Diversification of TAM receptor tyrosine kinase function. Nat Immunol. (2014) 15:920–8. doi: 10.1038/ni.2986 PMC4169336 25194421

[50] Rothlin CV Ghosh S Zuniga EI Oldstone MBA Lemke G. TAM receptors are pleiotropic inhibitors of the innate immune response. Cell. (2007) 131:1124–36. doi: 10.1016/j.cell.2007.10.034 18083102

[51] Gilchrist SE Goudarzi S Hafizi S. Gas6 inhibits toll-like receptor-mediated inflammatory pathways in mouse microglia via axl and mer. Front Cell Neurosci. (2020) 14:576650. doi: 10.3389/fncel.2020.576650 33192322 PMC7584110

[52] Dengler M Staufer K Huber H Stauber R Bantel H Weiss KH. Soluble Axl is an accurate biomarker of cirrhosis and hepatocellular carcinoma development: results from a large scale multicenter analysis. Oncotarget. (2017) 8:46234–48. doi: 10.18632/oncotarget.v8i28 PMC5542263 28526812

[53] Staufer K Dengler M Huber H Marculescu R Stauber R Lackner C. The non-invasive serum biomarker soluble Axl accurately detects advanced liver fibrosis and cirrhosis. Cell Death Dis. (2017) 8:e3135. doi: 10.1038/cddis.2017.554 29072690 PMC5680921

[54] Song X Wu A Ding Z Liang S Zhang C. Soluble axl is a novel diagnostic biomarker of hepatocellular carcinoma in chinese patients with chronic hepatitis B virus infection. Cancer Res Treat. (2020) 52:789–97. doi: 10.4143/crt.2019.749 PMC7373855 32138467

[55] Ortmayr G Brunnthaler L Pereyra D Huber H Santol J Rumpf B. Immunological aspects of AXL/GAS-6 in the context of human liver regeneration. Hepatol Commun. (2022) 6:576–92. doi: 10.1002/hep4.1832 PMC8870037 34951136

[56] Tran S Baba I Poupel L Dussaud S Moreau M Gélineau A. Impaired kupffer cell self-renewal alters the liver response to lipid overload during non-alcoholic steatohepatitis. Immunity. (2020) 53:627–640.e5. doi: 10.1016/j.immuni.2020.06.003 32562600

[57] Park MD Silvin A Ginhoux F Merad M. Macrophages in health and disease. Cell. (2022) 185:4259–79. doi: 10.1016/j.cell.2022.10.007 PMC9908006 36368305

[58] Brosseron F Maass A Kleineidam L Ravichandran KA Kolbe C-C Wolfsgruber S. Serum IL-6, sAXL, and YKL-40 as systemic correlates of reduced brain structure and function in Alzheimer’s disease: results from the DELCODE study. Alzheimers Res Ther. (2023) 15:13. doi: 10.1186/s13195-022-01118-0 36631909 PMC9835320

[59] Ko C-P Yu Y-L Hsiao P-C Yang S-F Yeh C-B. Plasma levels of soluble Axl correlate with severity of community-acquired pneumonia. Mol Med Rep. (2014) 9:1400–4. doi: 10.3892/mmr.2014.1933 24503651

[60] Batlle M Recarte-Pelz P Roig E Castel MA Cardona M Farrero M. AXL receptor tyrosine kinase is increased in patients with heart failure. Int J Cardiol. (2014) 173:402–9. doi: 10.1016/j.ijcard.2014.03.016 24681018

[61] Peiseler M Araujo David B Zindel J Surewaard BGJ Lee W-Y Heymann F. Kupffer cell-like syncytia replenish resident macrophage function in the fibrotic liver. Science. (2023) 381:eabq5202. doi: 10.1126/science.abq5202 37676943

[62] Miyao M Kotani H Ishida T Kawai C Manabe S Abiru H. Pivotal role of liver sinusoidal endothelial cells in NAFLD/NASH progression. Lab Invest. (2015) 95:1130–44. doi: 10.1038/labinvest.2015.95 26214582

[63] Gola A Dorrington MG Speranza E Sala C Shih RM Radtke AJ. Commensal-driven immune zonation of the liver promotes host defence. Nature. (2021) 589:131–6. doi: 10.1038/s41586-020-2977-2 PMC8691525 33239787

[64] Honda K Yanai H Negishi H Asagiri M Sato M Mizutani T. IRF-7 is the master regulator of type-I interferon-dependent immune responses. Nature. (2005) 434:772–7. doi: 10.1038/nature03464 15800576

[65] Guilliams M Bonnardel J Haest B Vanderborght B Wagner C Remmerie A. Spatial proteogenomics reveals distinct and evolutionarily conserved hepatic macrophage niches. Cell. (2022) 185:379–396.e38. doi: 10.1016/j.cell.2021.12.018 35021063 PMC8809252

[66] Maricic I Marrero I Eguchi A Nakamura R Johnson CD Dasgupta S. Differential activation of hepatic invariant NKT cell subsets plays a key role in progression of nonalcoholic steatohepatitis. J Immunol Baltim. Md 1950. (2018) 201:3017–35. doi: 10.4049/jimmunol.1800614 PMC6219905 30322964

[67] Hannibal TD Schmidt-Christensen A Nilsson J Fransén-Pettersson N Hansen L Holmberg D. Deficiency in plasmacytoid dendritic cells and type I interferon signalling prevents diet-induced obesity and insulin resistance in mice. Diabetologia. (2017) 60:2033–41. doi: 10.1007/s00125-017-4341-0 PMC6448810 28660492

[68] Garcia-Martinez I Santoro N Chen Y Hoque R Ouyang X Caprio S. Hepatocyte mitochondrial DNA drives nonalcoholic steatohepatitis by activation of TLR9. J Clin Invest. (2016) 126:859–64. doi: 10.1172/JCI83885 PMC4767345 26808498

[69] Swiecki M Wang Y Vermi W Gilfillan S Schreiber RD Colonna M. Type I interferon negatively controls plasmacytoid dendritic cell numbers *in vivo* . J Exp Med. (2011) 208:2367–74. doi: 10.1084/jem.20110654 PMC3256963 22084408

[70] Koda Y Teratani T Chu P-S Hagihara Y Mikami Y Harada Y. CD8+ tissue-resident memory T cells promote liver fibrosis resolution by inducing apoptosis of hepatic stellate cells. Nat Commun. (2021) 12:4474. doi: 10.1038/s41467-021-24734-0 34294714 PMC8298513

[71] Brenig R Pop OT Triantafyllou E Geng A Singanayagam A Perez-Shibayama C. Expression of AXL receptor tyrosine kinase relates to monocyte dysfunction and severity of cirrhosis. Life Sci Alliance. (2020) 3:e201900465. doi: 10.26508/lsa.201900465 31822557 PMC6907389

[72] Horst AK Tiegs G Diehl L. Contribution of macrophage efferocytosis to liver homeostasis and disease. Front Immunol. (2019) 10:2670. doi: 10.3389/fimmu.2019.02670 31798592 PMC6868070

