# Supplementary material for: Dynamic changes in immune cell populations by AXL kinase targeting diminish liver inflammation and fibrosis in experimental MASH
Source: Front Immunol. 2024 May 16;15:1400553. doi: 10.3389/fimmu.2024.1400553 (PMC11137289; doi:10.3389/fimmu.2024.1400553)
Supplement: Supplementary file 1 [file DataSheet_1.docx]

Supplementary Material

# Supplementary Figures and Tables

For more information on Supplementary Material and for details on the different file types accepted, please see [here](https://www.frontiersin.org/guidelines/author-guidelines#supplementary-material).

## Supplementary Figure 1


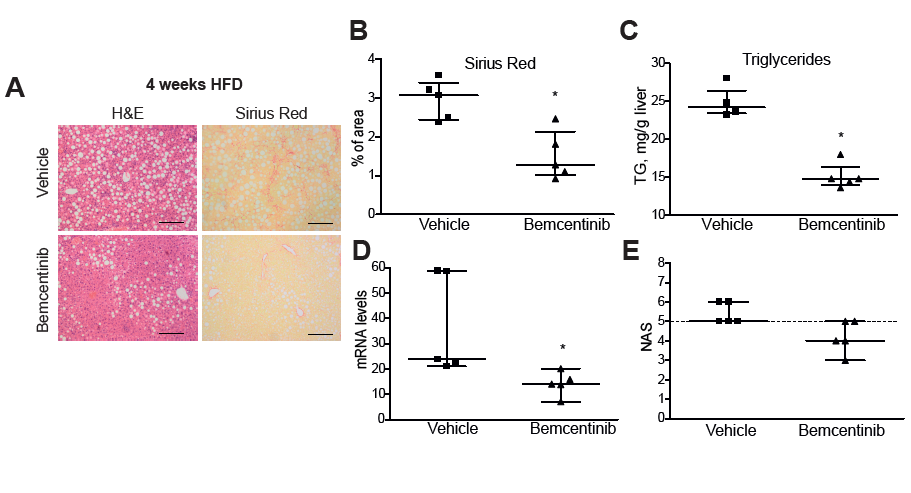


**Supplementary Figure 1. Bemcentinib ameliorates liver fibrosis and inflammation in early MASH.** Liver sections of mice fed HFD for 4 weeks and treated with vehicle (n=5) or bemcentinib 100mg/kg (n=5) for 2 weeks, while continuing on HFD, stained with H&E and Sirius Red (A), quantification of Sirius Red staining with ImageJ (B), triglycerides in liver extracts (C), mRNA expression level of Ccr2 in liver samples (D), and NAS (E) were assessed. Scale bar, 200 μm. H&E, hematoxylin and eosin; NAS, Nonalcoholic fatty liver disease activity score. Mann-Whitney test, *p ≤ .05 vs. vehicle.

## Supplementary Figure 2

##
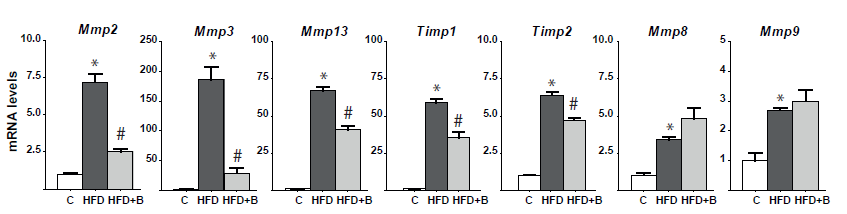


**Supplementary Figure 2. Bemcentinib promotes tissue remodeling.** Liver mRNA expression of matrix metalloproteinases (Mmps) and MMP inhibitors (Timps). One-way analysis of variance; *p≤.01 vs control diet (C), #p≤.01 vs. HFD; n=5/group.

## Supplementary Figure 3


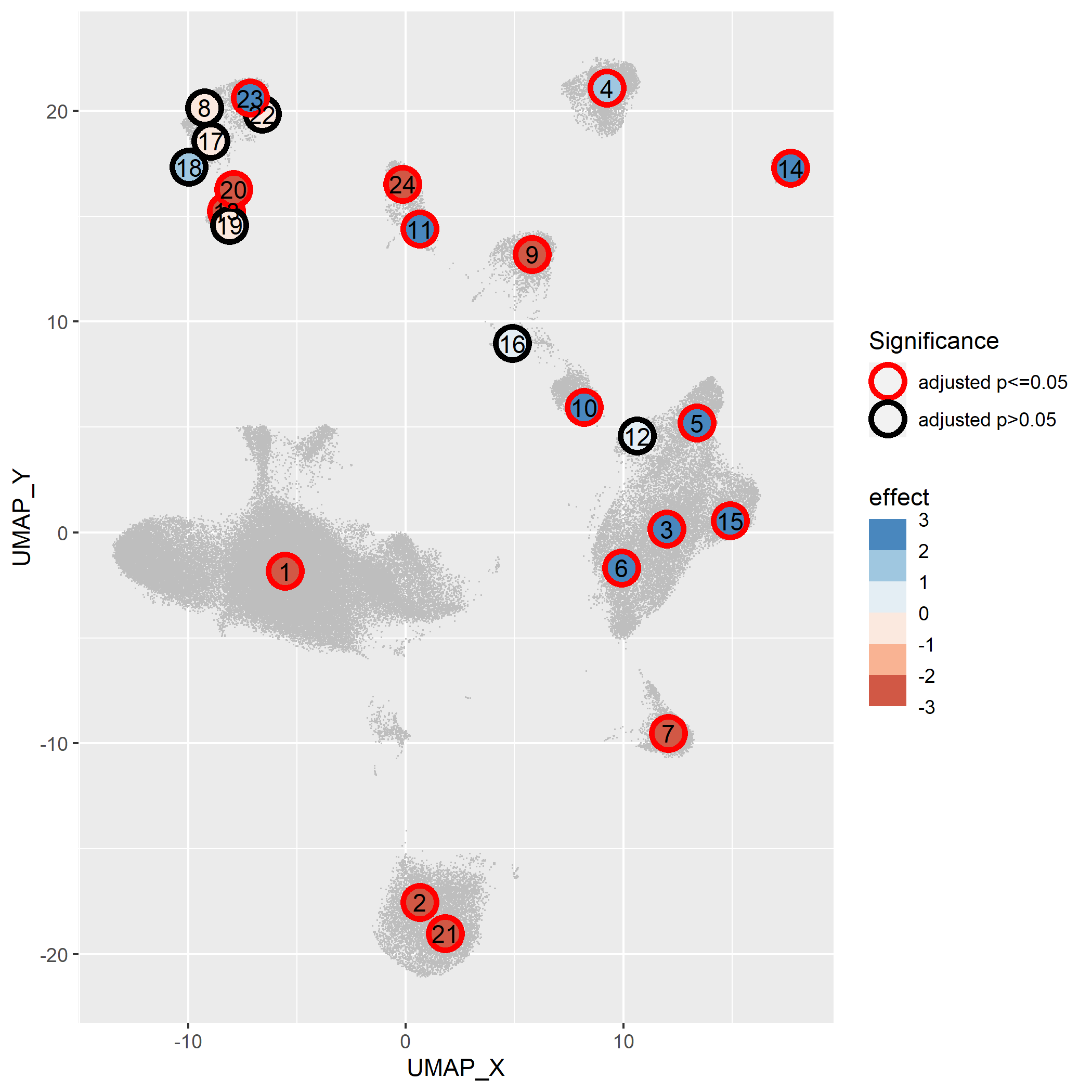


**Supplementary Figure 3.** Difference between chow and HFD fed mice. No bemcentinib treatment. Blue indicates increase and red indicates decrease in HFD fed mice.

## Supplementary Figure 4

**A**


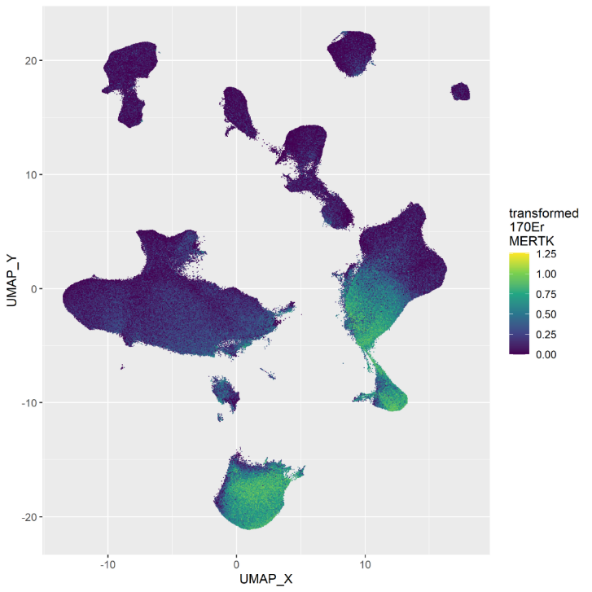


**B**

­­­­­­­
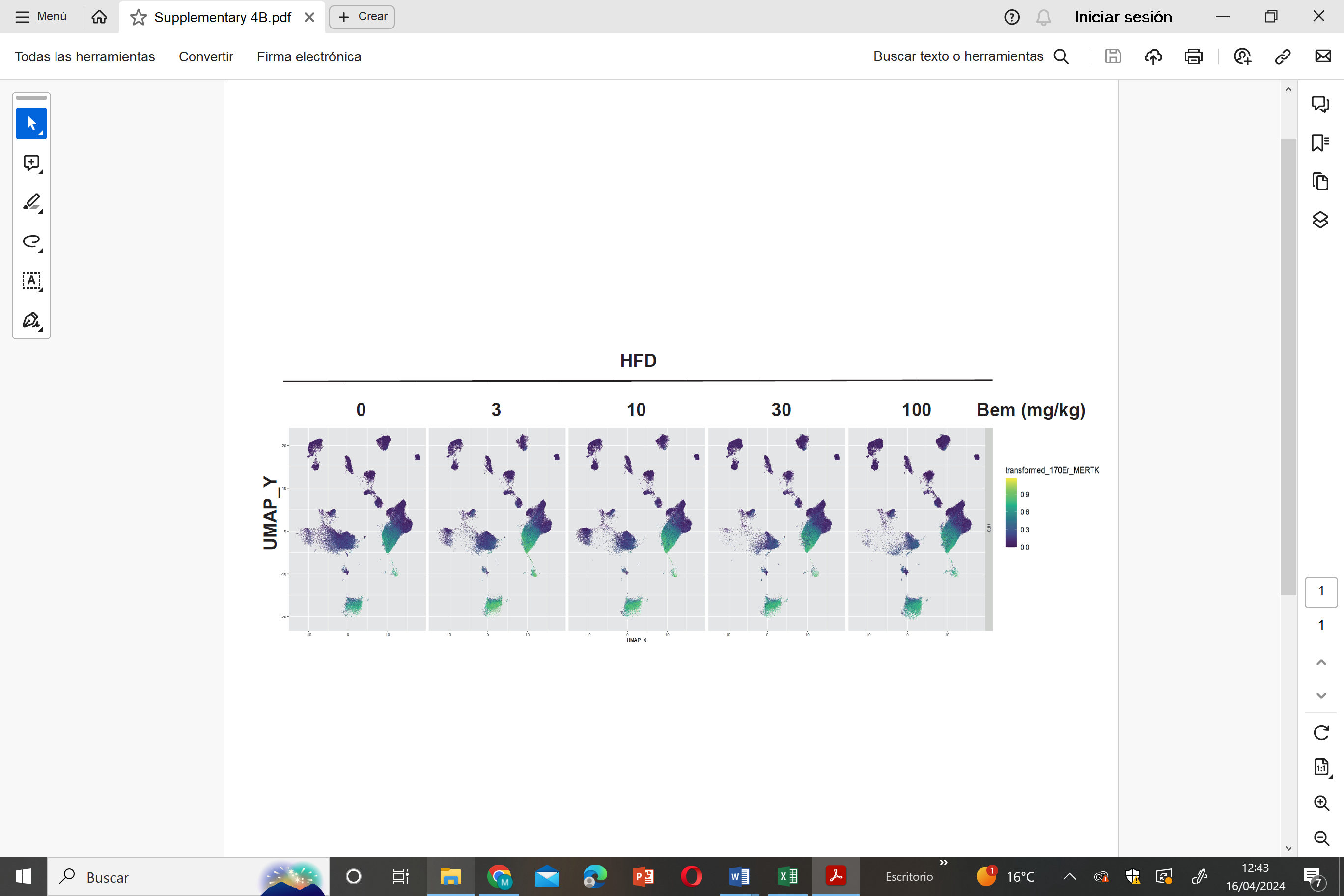


**Supplementary Figure 4.** A. UMAP of liver cells colored by MERTK signal intensity. B. UMAP of MERTK signal intensity in HFD-fed livers, 8 weeks, and bemcentinib (doses from 0 to 100 mg/kg) during the lasts two weeks.

## Supplementary Figure 5


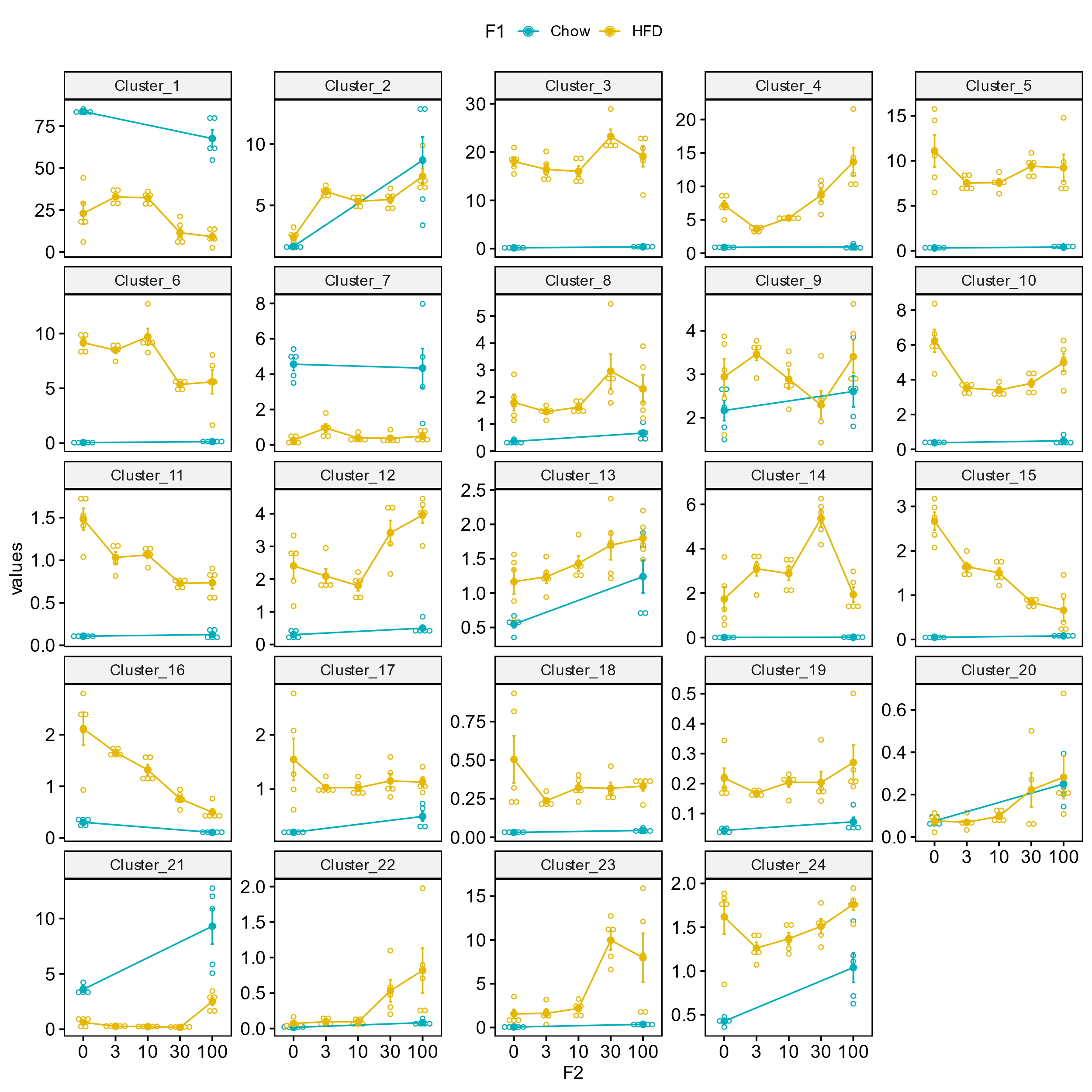


**Supplementary Figure 5.** Percentage each cluster makes up of total in each sample. X-axis is mg/kg bemcentinib, y-axis is percent, blue is samples from chow fed mice, yellow is samples from HFD fed mice.

## Supplementary Table 1

Supplementary table 1: antibody panel

| **Tag** | **Target** | **Clone** | **Vendor** | **Cat#** |
| --- | --- | --- | --- | --- |
| 89 | CD45 | 30-F11 | Fluidigm | 3089005B |
| 111 | CD117 | 2B8 | Thermo Fisher | 14-1171-85 |
| 112 | Ly-6A/E | D7 | Thermo Fisher | 14-5981-82 |
| 113 | CD163 | TNKUPJ | Thermo Fisher | 14-1631-82 |
| 114 | TCR gd | GL3 | Thermo Fisher | 14-5711-82 |
| 115 | B220 | RA3-6B2 | Thermo Fisher | 14-0452-85 |
| 116 | TCR beta | H57-597 | Thermo Fisher | 14-5961-82 |
| 141 | Ly-6G | 1A8 | Fluidigm | 3141008B |
| 142 | CD11c | N418 | Thermo Fisher | 14-0114-85 |
| 143 | CD71 | R17217 | Thermo Fisher | 14-0711-82 |
| 144 | CD115 | AFS98 | Fluidigm | 3144012B |
| 145 | CD4 | RM4-5 | Fluidigm | 3145002B |
| 146 | CD8a | 53-6.7 | Fluidigm | 3146003B |
| 147 | TIM4 | RMT4-54 | BioLegend | 130002 |
| 148 | CD169 | 3D6.112 | BioLegend | 142402 |
| 149 | CD83 | Michel-17 | Thermo Fisher | 14-0831-82 |
| 150 | CD24 | M1/69 | Fluidigm | 3150009B |
| 151 | CD64 | X54-5/7.1 | Fluidigm | 3151012B |
| 152 | CD3e | 145-2C11 | Fluidigm | 3152004B |
| 153 | PD-L1 | 10F.9G2 | Fluidigm | 3153016B |
| 154 | CD11b | M1/70 | Fluidigm | 3154006B |
| 155 | CD90.2 | 53-2.1 | Thermo Fisher | 14-0902-82 |
| 156 | CD103 | 2e7 | Thermo Fisher | 14-1031-85 |
| 157 | CD170 | 1RNM44N | Thermo Fisher | 14-1702-82 |
| 158 | Foxp3 | FJK-16s | Fluidigm | 3158003A |
| 159 | F4/80 | BM8 | Fluidigm | 3159009B |
| 160 | Arg1 | Polyclonal | Novus | NBP1-32731 |
| 161 | GrB | GB11 | Thermo Fisher | MA1-80734 |
| 162 | Ly6C | HK1.4 | Fluidigm | 3162014B |
| 163 | CD73 | TY/11.8 | BioLegend | 127202 |
| 164 | CD49b | HMa2 | Fluidigm | 3164011B |
| 165 | CX3CR1 | SA011F11 | BioLegend | 149002 |
| 167 | CD335 | 29A1.4 | Fluidigm | 3167008B |
| 169 | CD206 | C068C2 | Fluidigm | 3169021B |
| 170 | MerTK | 2B10C42 | BioLegend | 151502 |
| 170 | MerTK | DS5MMER | Thermo Fisher | 14-5751-82 |
| 171 | CD80 | 16-10A1 | Fluidigm | 3171008B |
| 172 | CD86 | GL1 | Fluidigm | 3172016B |
| 173 | TGFb | TW7-16B4 | BioLegend | 141402 |
| 175 | Gas6 | Polyclonal | R&D | AF986 |
| 176 | Axl | 175128 | R&D | MAB8541 |
| 176 | Axl | 107332 | R&D | MAB854 |
| 176 | Axl | 107332 | R&D | MAB854 |
| 209 | I-A/I-E | M5/114.15.2 | Fluidigm | 3209006B |

## Supplementary Table 2

Supplementary table 2: Percentage each cluster makes up in every sample.

|  | Cluster_1 | Cluster_2 | Cluster_3 | Cluster_4 | Cluster_5 | Cluster_6 | Cluster_7 | Cluster_8 | Cluster_9 | Cluster_10 | Cluster_11 | Cluster_12 | Cluster_13 | Cluster_14 | Cluster_15 | Cluster_16 | Cluster_17 | Cluster_18 | Cluster_19 | Cluster_20 | Cluster_21 | Cluster_22 | Cluster_23 | Cluster_24 | F1 | F2 |
| --- | --- | --- | --- | --- | --- | --- | --- | --- | --- | --- | --- | --- | --- | --- | --- | --- | --- | --- | --- | --- | --- | --- | --- | --- | --- | --- |
| Chow_0mg_1.fcs | 83.7 | 1.5 | 0.2 | 1.1 | 0.4 | 0.0 | 3.9 | 0.4 | 2.7 | 0.3 | 0.1 | 0.4 | 0.7 | 0.0 | 0.0 | 0.2 | 0.2 | 0.0 | 0.1 | 0.1 | 3.5 | 0.0 | 0.1 | 0.4 | Chow | 0mg |
| Chow_0mg_2.fcs | 84.6 | 1.8 | 0.1 | 0.9 | 0.3 | 0.0 | 3.5 | 0.4 | 2.6 | 0.3 | 0.1 | 0.2 | 0.6 | 0.0 | 0.0 | 0.3 | 0.2 | 0.0 | 0.0 | 0.1 | 3.4 | 0.0 | 0.0 | 0.4 | Chow | 0mg |
| Chow_0mg_3.fcs | 82.7 | 1.6 | 0.2 | 0.9 | 0.4 | 0.1 | 4.9 | 0.4 | 2.2 | 0.5 | 0.1 | 0.5 | 0.6 | 0.0 | 0.1 | 0.3 | 0.2 | 0.0 | 0.0 | 0.1 | 3.7 | 0.0 | 0.1 | 0.4 | Chow | 0mg |
| Chow_0mg_4.fcs | 85.2 | 1.4 | 0.2 | 0.7 | 0.2 | 0.0 | 5.1 | 0.3 | 1.5 | 0.3 | 0.1 | 0.2 | 0.4 | 0.0 | 0.1 | 0.4 | 0.2 | 0.0 | 0.0 | 0.1 | 3.2 | 0.0 | 0.1 | 0.4 | Chow | 0mg |
| Chow_0mg_5.fcs | 82.3 | 1.9 | 0.2 | 0.8 | 0.2 | 0.1 | 5.4 | 0.4 | 1.8 | 0.4 | 0.1 | 0.3 | 0.6 | 0.0 | 0.1 | 0.3 | 0.2 | 0.0 | 0.0 | 0.1 | 4.2 | 0.0 | 0.1 | 0.5 | Chow | 0mg |
| Chow_100mg_1.fcs | 79.8 | 3.4 | 0.2 | 0.9 | 0.4 | 0.1 | 3.3 | 0.4 | 2.6 | 0.3 | 0.1 | 0.4 | 0.7 | 0.0 | 0.1 | 0.1 | 0.3 | 0.0 | 0.0 | 0.1 | 5.9 | 0.0 | 0.2 | 0.6 | Chow | 100mg |
| Chow_100mg_2.fcs | 62.6 | 8.8 | 0.5 | 0.8 | 0.3 | 0.2 | 8.0 | 0.5 | 1.8 | 0.5 | 0.1 | 0.4 | 0.7 | 0.0 | 0.1 | 0.1 | 0.3 | 0.0 | 0.1 | 0.2 | 12.7 | 0.0 | 0.5 | 0.7 | Chow | 100mg |
| Chow_100mg_3.fcs | 54.8 | 12.9 | 0.6 | 1.4 | 0.7 | 0.2 | 5.0 | 1.1 | 3.9 | 0.8 | 0.2 | 0.9 | 1.9 | 0.0 | 0.1 | 0.2 | 0.7 | 0.1 | 0.1 | 0.4 | 12.0 | 0.1 | 0.4 | 1.6 | Chow | 100mg |
| Chow_100mg_4.fcs | 61.0 | 12.9 | 0.4 | 1.0 | 0.4 | 0.1 | 4.3 | 0.7 | 2.7 | 0.5 | 0.2 | 0.4 | 1.7 | 0.0 | 0.1 | 0.1 | 0.6 | 0.1 | 0.1 | 0.2 | 10.9 | 0.1 | 0.4 | 1.2 | Chow | 100mg |
| Chow_100mg_5.fcs | 79.8 | 5.5 | 0.2 | 0.6 | 0.2 | 0.0 | 1.2 | 0.7 | 2.0 | 0.3 | 0.1 | 0.4 | 1.2 | 0.0 | 0.0 | 0.1 | 0.5 | 0.0 | 0.1 | 0.3 | 5.1 | 0.1 | 0.3 | 1.1 | Chow | 100mg |
| HFD_0mg_1.fcs | 16.8 | 1.5 | 16.9 | 8.5 | 14.5 | 9.4 | 0.1 | 1.7 | 3.7 | 6.6 | 1.7 | 2.8 | 1.6 | 2.3 | 3.0 | 2.8 | 2.1 | 0.8 | 0.2 | 0.1 | 0.3 | 0.0 | 0.7 | 1.8 | HFD | 0mg |
| HFD_0mg_2.fcs | 6.0 | 2.1 | 20.9 | 8.7 | 15.8 | 9.9 | 0.0 | 2.0 | 3.1 | 8.4 | 1.7 | 3.3 | 1.3 | 3.6 | 2.8 | 2.4 | 2.8 | 0.9 | 0.2 | 0.1 | 0.2 | 0.2 | 1.7 | 1.9 | HFD | 0mg |
| HFD_0mg_3.fcs | 19.2 | 3.2 | 18.6 | 7.1 | 10.6 | 8.3 | 0.4 | 2.8 | 3.9 | 5.9 | 1.5 | 2.8 | 1.4 | 1.3 | 2.1 | 2.4 | 1.3 | 0.2 | 0.3 | 0.1 | 1.1 | 0.1 | 3.5 | 1.8 | HFD | 0mg |
| HFD_0mg_4.fcs | 44.2 | 2.7 | 15.5 | 5.0 | 6.5 | 8.4 | 0.2 | 1.1 | 1.6 | 4.3 | 1.0 | 1.2 | 0.6 | 0.9 | 2.4 | 0.9 | 0.6 | 0.2 | 0.2 | 0.0 | 0.8 | 0.0 | 0.9 | 0.8 | HFD | 0mg |
| HFD_0mg_5.fcs | 29.0 | 2.5 | 18.3 | 6.7 | 8.2 | 9.9 | 0.6 | 1.3 | 2.4 | 6.0 | 1.4 | 1.9 | 0.9 | 0.6 | 3.2 | 2.1 | 1.0 | 0.3 | 0.2 | 0.1 | 0.7 | 0.1 | 1.0 | 1.8 | HFD | 0mg |
| HFD_100mg_1.fcs | 13.3 | 6.9 | 22.9 | 14.3 | 7.6 | 7.1 | 0.3 | 1.2 | 2.9 | 6.3 | 0.9 | 4.0 | 1.6 | 1.3 | 1.5 | 0.8 | 0.9 | 0.4 | 0.2 | 0.2 | 1.6 | 0.2 | 1.8 | 1.8 | HFD | 100mg |
| HFD_100mg_2.fcs | 7.6 | 9.9 | 20.8 | 10.6 | 7.2 | 8.1 | 0.9 | 1.8 | 4.6 | 4.4 | 0.6 | 4.1 | 1.7 | 1.5 | 0.3 | 0.4 | 1.0 | 0.2 | 0.2 | 0.1 | 3.5 | 0.9 | 8.3 | 1.5 | HFD | 100mg |
| HFD_100mg_3.fcs | 2.5 | 6.4 | 18.2 | 10.1 | 14.8 | 1.6 | 0.2 | 3.1 | 2.9 | 5.7 | 0.6 | 4.3 | 1.5 | 2.5 | 0.4 | 0.5 | 1.2 | 0.3 | 0.2 | 0.2 | 3.1 | 2.0 | 15.9 | 1.9 | HFD | 100mg |
| HFD_100mg_4.fcs | 14.1 | 7.2 | 22.7 | 11.8 | 9.9 | 5.7 | 0.4 | 1.5 | 2.7 | 5.2 | 0.8 | 3.0 | 2.2 | 3.0 | 1.0 | 0.4 | 1.1 | 0.4 | 0.2 | 0.2 | 2.7 | 0.3 | 1.7 | 1.8 | HFD | 100mg |
| HFD_100mg_5.fcs | 8.2 | 6.5 | 11.1 | 21.6 | 6.7 | 5.5 | 0.7 | 3.9 | 3.9 | 3.4 | 0.8 | 4.5 | 2.0 | 1.4 | 0.2 | 0.4 | 1.4 | 0.4 | 0.5 | 0.7 | 1.7 | 0.7 | 12.1 | 1.7 | HFD | 100mg |
| HFD_10mg_1.fcs | 30.0 | 5.5 | 18.6 | 5.0 | 8.8 | 9.2 | 0.7 | 1.8 | 2.7 | 3.1 | 1.1 | 1.5 | 1.9 | 2.2 | 1.4 | 1.2 | 1.2 | 0.2 | 0.2 | 0.1 | 0.3 | 0.1 | 1.5 | 1.5 | HFD | 10mg |
| HFD_10mg_2.fcs | 33.0 | 5.8 | 14.7 | 5.4 | 7.3 | 8.3 | 0.2 | 1.4 | 3.2 | 3.3 | 1.1 | 2.0 | 1.4 | 3.4 | 1.2 | 1.6 | 1.1 | 0.4 | 0.2 | 0.1 | 0.2 | 0.1 | 3.2 | 1.2 | HFD | 10mg |
| HFD_10mg_3.fcs | 34.8 | 4.8 | 13.8 | 5.1 | 7.6 | 9.3 | 0.3 | 1.5 | 3.5 | 3.5 | 1.2 | 2.2 | 1.4 | 3.1 | 1.7 | 1.6 | 0.9 | 0.4 | 0.1 | 0.1 | 0.2 | 0.1 | 1.2 | 1.5 | HFD | 10mg |
| HFD_10mg_4.fcs | 36.1 | 5.0 | 14.2 | 5.5 | 6.3 | 9.0 | 0.3 | 1.9 | 2.7 | 3.3 | 0.9 | 1.9 | 1.2 | 3.6 | 1.4 | 1.2 | 0.9 | 0.3 | 0.2 | 0.1 | 0.2 | 0.0 | 2.2 | 1.3 | HFD | 10mg |
| HFD_10mg_5.fcs | 27.5 | 5.5 | 18.8 | 5.4 | 7.8 | 12.7 | 0.4 | 1.5 | 2.2 | 3.9 | 1.0 | 1.4 | 1.3 | 2.1 | 1.8 | 1.1 | 0.9 | 0.3 | 0.2 | 0.1 | 0.2 | 0.1 | 2.7 | 1.3 | HFD | 10mg |
| HFD_30mg_1.fcs | 16.0 | 5.8 | 21.3 | 10.1 | 8.4 | 5.0 | 0.9 | 2.7 | 3.4 | 3.3 | 0.8 | 4.1 | 1.7 | 4.9 | 0.9 | 0.8 | 1.0 | 0.2 | 0.2 | 0.1 | 0.3 | 0.2 | 6.6 | 1.4 | HFD | 30mg |
| HFD_30mg_2.fcs | 8.4 | 5.7 | 21.8 | 10.9 | 9.8 | 5.7 | 0.3 | 5.5 | 2.3 | 3.7 | 0.7 | 2.2 | 1.9 | 4.2 | 0.8 | 0.7 | 1.6 | 0.3 | 0.3 | 0.3 | 0.2 | 0.5 | 11.0 | 1.3 | HFD | 30mg |
| HFD_30mg_3.fcs | 6.4 | 4.8 | 23.2 | 9.1 | 10.9 | 5.7 | 0.1 | 2.2 | 2.4 | 4.3 | 0.8 | 3.5 | 1.3 | 6.3 | 0.9 | 0.9 | 1.3 | 0.3 | 0.2 | 0.5 | 0.1 | 0.6 | 12.7 | 1.6 | HFD | 30mg |
| HFD_30mg_4.fcs | 21.2 | 4.8 | 21.0 | 5.8 | 8.2 | 5.5 | 0.3 | 2.7 | 1.9 | 3.3 | 0.7 | 4.2 | 1.2 | 5.9 | 0.9 | 0.8 | 1.0 | 0.3 | 0.2 | 0.1 | 0.1 | 0.3 | 8.1 | 1.5 | HFD | 30mg |
| HFD_30mg_5.fcs | 5.6 | 6.4 | 28.9 | 7.6 | 9.7 | 4.8 | 0.2 | 1.8 | 1.4 | 4.4 | 0.7 | 3.1 | 2.4 | 5.6 | 0.7 | 0.5 | 0.9 | 0.5 | 0.1 | 0.2 | 0.1 | 1.1 | 11.4 | 1.8 | HFD | 30mg |
| HFD_3mg_1.fcs | 32.9 | 6.4 | 15.7 | 3.8 | 7.0 | 9.0 | 1.0 | 1.6 | 3.6 | 3.7 | 1.0 | 1.8 | 1.2 | 3.6 | 1.6 | 1.7 | 1.0 | 0.2 | 0.2 | 0.0 | 0.2 | 0.1 | 1.4 | 1.2 | HFD | 3mg |
| HFD_3mg_2.fcs | 29.0 | 5.9 | 17.5 | 3.4 | 8.6 | 7.5 | 1.8 | 1.4 | 3.4 | 3.3 | 0.8 | 3.0 | 1.5 | 3.7 | 1.5 | 1.7 | 1.0 | 0.2 | 0.2 | 0.1 | 0.1 | 0.2 | 3.2 | 1.1 | HFD | 3mg |
| HFD_3mg_3.fcs | 36.3 | 5.7 | 14.5 | 3.8 | 6.9 | 8.7 | 0.5 | 1.7 | 3.8 | 3.1 | 1.2 | 1.9 | 0.9 | 3.3 | 1.5 | 1.6 | 1.0 | 0.3 | 0.2 | 0.1 | 0.4 | 0.1 | 1.3 | 1.4 | HFD | 3mg |
| HFD_3mg_4.fcs | 37.4 | 6.1 | 14.3 | 3.9 | 6.9 | 8.9 | 0.5 | 1.1 | 2.9 | 3.7 | 1.2 | 1.8 | 1.2 | 3.0 | 2.0 | 1.6 | 1.0 | 0.2 | 0.2 | 0.1 | 0.3 | 0.0 | 0.3 | 1.4 | HFD | 3mg |
| HFD_3mg_5.fcs | 28.9 | 6.6 | 20.1 | 3.1 | 8.2 | 8.4 | 1.0 | 1.5 | 3.7 | 3.7 | 1.0 | 2.1 | 1.3 | 1.9 | 1.6 | 1.7 | 1.2 | 0.2 | 0.2 | 0.1 | 0.2 | 0.1 | 1.9 | 1.2 | HFD | 3mg |

## Supplementary Table 3

Supplementary table 3: Aldex2 derived statistical results comparing HFD 0mg to HFD 100mg. rab=relative abundance, diff=difference, btw=between, win=within, we=welch, ep=expected p-value, wi=Wilcoxon rank sum, eBH=expected Benjanimini Hochberg.

|  | rab.all | rab.win.HFD_0mg | rab.win.HFD_100mg | diff.btw | diff.win | effect | overlap | we.ep | we.eBH | wi.ep | wi.eBH |
| --- | --- | --- | --- | --- | --- | --- | --- | --- | --- | --- | --- |
| Cluster_1 | 2.887569 | 3.368254 | 2.009252 | -1.38316 | 1.577744 | -0.88812 | 0.165625 | 0.066592 | 0.116513 | 0.095238 | 0.143458 |
| Cluster_2 | 1.457337 | 0.743284 | 1.914132 | 1.194201 | 0.525446 | 2.290363 | 0.000219 | 0.002284 | 0.016093 | 0.007937 | 0.023299 |
| Cluster_3 | 3.503167 | 3.586007 | 3.463663 | -0.12755 | 0.329916 | -0.24009 | 0.333333 | 0.338492 | 0.419556 | 0.522693 | 0.619153 |
| Cluster_4 | 2.402016 | 2.18307 | 2.652122 | 0.535662 | 0.385671 | 1.404081 | 0.003147 | 0.022765 | 0.065465 | 0.007999 | 0.02341 |
| Cluster_5 | 2.468994 | 2.563892 | 2.060694 | -0.5768 | 0.642543 | -0.89859 | 0.121876 | 0.083719 | 0.134526 | 0.055556 | 0.099447 |
| Cluster_6 | 2.129885 | 2.526651 | 1.587838 | -0.95329 | 0.573839 | -1.54963 | 0.000219 | 0.040557 | 0.087956 | 0.007937 | 0.023299 |
| Cluster_7 | -2.26276 | -2.3117 | -2.221 | 0.655632 | 1.889849 | 0.367259 | 0.358256 | 0.307979 | 0.398798 | 0.521825 | 0.619652 |
| Cluster_8 | 0.052614 | 0.073879 | -0.07445 | -0.13342 | 0.740291 | -0.18642 | 0.440625 | 0.950173 | 0.956587 | 0.840092 | 0.879215 |
| Cluster_9 | 0.741239 | 0.84127 | 0.692707 | -0.10458 | 0.383113 | -0.26481 | 0.4 | 0.747112 | 0.840578 | 0.728981 | 0.789093 |
| Cluster_10 | 1.714029 | 1.992575 | 1.463147 | -0.53173 | 0.395468 | -1.25723 | 0.040501 | 0.027558 | 0.07023 | 0.017857 | 0.043332 |
| Cluster_11 | -0.64607 | -0.07526 | -1.38601 | -1.31708 | 0.336967 | -3.9864 | 0.000219 | 0.000375 | 0.004497 | 0.007937 | 0.023299 |
| Cluster_12 | 0.838755 | 0.56226 | 1.113384 | 0.489124 | 0.424262 | 1.223628 | 0.081251 | 0.028242 | 0.071152 | 0.031808 | 0.068402 |
| Cluster_13 | -0.22238 | -0.40819 | -0.13688 | 0.340054 | 0.390357 | 0.955078 | 0.16511 | 0.059083 | 0.107774 | 0.102431 | 0.152679 |
| Cluster_14 | -0.33679 | -0.31921 | -0.35281 | 0.007657 | 1.054854 | 0.010433 | 0.49375 | 0.87929 | 0.923587 | 0.991319 | 0.991891 |
| Cluster_15 | -0.08012 | 0.855975 | -2.2581 | -2.98912 | 1.289353 | -2.04321 | 0.000219 | 0.007206 | 0.034498 | 0.007937 | 0.023299 |
| Cluster_16 | -0.74129 | 0.448886 | -2.12737 | -2.53309 | 0.602344 | -3.9968 | 0.000219 | 1.72E-05 | 0.000412 | 0.007937 | 0.023299 |
| Cluster_17 | -0.62215 | -0.54803 | -0.81182 | -0.30245 | 0.453922 | -0.76042 | 0.156251 | 0.116204 | 0.17433 | 0.087674 | 0.135166 |
| Cluster_18 | -2.34002 | -2.21994 | -2.43207 | -0.41441 | 0.886402 | -0.7122 | 0.203125 | 0.176743 | 0.24952 | 0.163504 | 0.229966 |
| Cluster_19 | -3.01743 | -2.88306 | -3.12599 | -0.12377 | 0.534941 | -0.20364 | 0.384375 | 0.832734 | 0.885011 | 0.629898 | 0.697648 |
| Cluster_20 | -4.03856 | -4.37569 | -3.16398 | 1.29866 | 1.214387 | 1.228168 | 0.050002 | 0.020047 | 0.061137 | 0.024058 | 0.051373 |
| Cluster_21 | -0.41237 | -1.08104 | 0.544519 | 1.641778 | 1.19411 | 1.536751 | 0.000219 | 0.016185 | 0.058579 | 0.007937 | 0.023299 |
| Cluster_22 | -3.10432 | -4.97767 | -1.5708 | 3.198407 | 1.5857 | 2.080825 | 0.003147 | 0.002729 | 0.016893 | 0.008061 | 0.023528 |
| Cluster_23 | 0.001601 | -0.30013 | 2.138153 | 2.155603 | 2.086074 | 1.033199 | 0.133957 | 0.054232 | 0.104227 | 0.060268 | 0.102248 |
| Cluster_24 | -0.04125 | 0.100614 | -0.05789 | -0.16773 | 0.28678 | -0.49405 | 0.325 | 0.363193 | 0.446368 | 0.420201 | 0.525179 |

## Supplementary Table 4

Supplementary table 4: Aldex2 derived statistical results comparing Chow 0mg to Chow 100mg. rab=relative abundance, diff=difference, btw=between, win=within, we=welch, ep=expected p-value, wi=Wilcoxon rank sum, eBH=expected Benjanimini Hochberg.

|  | rab.all | rab.win.Chow_0mg | rab.win.Chow_100mg | diff.btw | diff.win | effect | overlap | we.ep | we.eBH | wi.ep | wi.eBH |
| --- | --- | --- | --- | --- | --- | --- | --- | --- | --- | --- | --- |
| Cluster_1 | 8.014104 | 8.314146 | 7.146316 | -1.15355 | 0.764759 | -1.59509 | 0.000219 | 0.016007 | 0.046843 | 0.007937 | 0.028134 |
| Cluster_2 | 3.128945 | 2.634312 | 4.251656 | 1.450269 | 0.439185 | 3.163387 | 0.000219 | 0.001051 | 0.007951 | 0.007937 | 0.028134 |
| Cluster_3 | -0.56121 | -0.65161 | -0.47722 | 0.190332 | 0.434506 | 0.42659 | 0.328125 | 0.432108 | 0.520924 | 0.445995 | 0.543477 |
| Cluster_4 | 1.522516 | 1.61239 | 0.919928 | -0.71218 | 0.39899 | -1.89825 | 0.009386 | 0.003418 | 0.016737 | 0.008929 | 0.030236 |
| Cluster_5 | 0.082718 | 0.206798 | -0.42763 | -0.54427 | 0.652391 | -0.83569 | 0.208723 | 0.122554 | 0.194249 | 0.164869 | 0.252513 |
| Cluster_6 | -2.38051 | -2.43836 | -1.51111 | 0.821553 | 0.910419 | 0.659972 | 0.35514 | 0.293516 | 0.391554 | 0.499504 | 0.596576 |
| Cluster_7 | 3.879094 | 4.008499 | 2.970683 | -1.02484 | 0.86793 | -1.14447 | 0.124611 | 0.050318 | 0.098535 | 0.055556 | 0.101891 |
| Cluster_8 | 0.425053 | 0.474212 | 0.387707 | 0.018793 | 0.369773 | 0.053688 | 0.478125 | 0.859908 | 0.882913 | 0.931114 | 0.943002 |
| Cluster_9 | 2.70941 | 2.879009 | 2.435612 | -0.47092 | 0.560159 | -1.10786 | 0.115266 | 0.04088 | 0.089658 | 0.055556 | 0.101891 |
| Cluster_10 | 0.279118 | 0.547533 | -0.10507 | -0.51917 | 0.32211 | -1.64199 | 0.053127 | 0.009933 | 0.034557 | 0.021329 | 0.058179 |
| Cluster_11 | -1.4844 | -1.31233 | -1.92306 | -0.61186 | 0.332056 | -1.87714 | 0.003156 | 0.010425 | 0.035216 | 0.009487 | 0.031392 |
| Cluster_12 | 0.052125 | -0.00678 | 0.081642 | -0.05205 | 0.552728 | -0.08512 | 0.461059 | 0.805714 | 0.845056 | 0.93006 | 0.942325 |
| Cluster_13 | 1.176037 | 1.12222 | 1.477477 | 0.330043 | 0.491736 | 0.678647 | 0.259375 | 0.257505 | 0.360469 | 0.290117 | 0.401252 |
| Cluster_14 | -5.24441 | -5.29297 | -5.13057 | 0.262003 | 1.03922 | 0.226334 | 0.429907 | 0.78313 | 0.822988 | 0.705419 | 0.763588 |
| Cluster_15 | -2.49849 | -2.4333 | -2.6058 | -0.17931 | 0.427037 | -0.35784 | 0.345795 | 0.422027 | 0.508157 | 0.488343 | 0.579727 |
| Cluster_16 | -1.20402 | 0.194513 | -2.1506 | -2.34407 | 0.466667 | -5.06895 | 0.000219 | 0.000134 | 0.001725 | 0.007937 | 0.028134 |
| Cluster_17 | -0.17984 | -0.38262 | 0.119471 | 0.418238 | 0.400088 | 1.012262 | 0.146876 | 0.064348 | 0.115595 | 0.099516 | 0.165985 |
| Cluster_18 | -3.31539 | -3.10192 | -3.43574 | -0.29187 | 0.580021 | -0.43892 | 0.317757 | 0.322315 | 0.411543 | 0.438182 | 0.530369 |
| Cluster_19 | -2.69966 | -2.56997 | -2.78934 | -0.16994 | 0.400457 | -0.39961 | 0.334375 | 0.428744 | 0.509352 | 0.487537 | 0.571071 |
| Cluster_20 | -1.3385 | -1.87046 | -1.11277 | 0.846798 | 0.408024 | 2.042546 | 0.000219 | 0.002354 | 0.012806 | 0.007937 | 0.028134 |
| Cluster_21 | 3.89088 | 3.786914 | 4.177323 | 0.421967 | 0.394326 | 1.117622 | 0.140188 | 0.056389 | 0.103953 | 0.059462 | 0.107718 |
| Cluster_22 | -3.24188 | -4.45595 | -2.80524 | 1.751061 | 1.377695 | 1.279564 | 0.075001 | 0.019226 | 0.052816 | 0.026166 | 0.066619 |
| Cluster_23 | -1.33028 | -2.3244 | -0.47028 | 1.858734 | 0.57372 | 3.250801 | 0.000219 | 8.52E-05 | 0.00144 | 0.007937 | 0.028134 |
| Cluster_24 | 0.806514 | 0.732137 | 1.113545 | 0.397927 | 0.309967 | 1.157635 | 0.118751 | 0.048215 | 0.095977 | 0.057478 | 0.105624 |

# Supplementary methods

*Determination of soluble AXL (sAXL) levels.* sAXL levels were determined in mouse serum samples by specific sandwich ELISA using commercial kits (DuoSet ELISA, R&D). Briefly, for 96-well plate preparation, the capture antibody was diluted according to the manufacturer’s protocol and 100µl were added to each ELISA plate well. Seal the plate and incubate overnight at room temperature. Wash plate 4 times and block with 300µl blocking buffer/well for 1h at R.T. Wash again 4 times. Prepare serial dilution standard from in the appropriate diluent. Add 100µl of standard or sample to each well in triplicate. Incubate at room temperature for at least 2 hours. Wash plate four times. Dilute detection antibody (biotinylated) in diluent to a concentration of 0.5µg/mL (500ng/ml). Immediately add 100µl per well. Incubate at room temperature for 2 hours. Aspirate and wash plate 4 times. Dilute Streptavidin-HRP conjugate 1:20,000 in diluent. Add 100µl per well. Incubate 30 min at room temperature. Aspirate and wash plate 4 times. Add 100µl of substrate solution (TMB liquid substrate) to each well. Incubate at room temperature for 20 minutes. Add 100µl 1M HCl stop solution to each well. Monitor color development with an ELISA plate reader at 450 nm with wavelength correction set at 620 nm.

*Tissue digestion protocol for CYTOF.* After anesthesia with sodium pentobarbital (50 mg/kg), mice were opened, a blood sample was taken, and the liver was harvested and transferred to 15 mL tube with 3 mL MACS tissue storage (Miltenyi Biotech) medium on ice. To digest the liver, we used the Multi tissue digestion kit-1 (MTK1, Miltenyi Biotech). To prepare 5 mL digestion solution: add 4.7 mL RPMI, 200 µl Enzyme D, 100 µl of Enzyme R, and 25 µL of Enzyme A. Keep at 37 °C. Transfer the liver to a petri dish (after filtering through sterile gauze) and add the digestion solution. Cut the tissue into small pieces (1-2mm pieces) using a scalpel or scissors. Transfer minced liver and digestion solution to a 15 mL tube. Put tube in a rotation wheel at 37 °C for 30 min. Afterwards, add 8 mL RPMI 1640 (10% FBS 1% DNase) to the 15 mL -tube. Then filter the solution into a 15 mL tube, through a 100µm filter. Spin down at 300g 5min RT and aspirate supernatant. To lyse the remaining red blood cell, resuspend pellet in 5 mL 1X RBC-lysis buffer (Miltenyi Biotech) and incubate for 5 minutes RT. Centrifuge at 300g for 5 min RT and remove supernatant. Resuspend in 10 mL RPMI1640 (10% FBS 1% DNase) and incubate at RT for 10 min. pin down at 300g 5min. To stain Live vs dead cells: resuspend cells in 500 µl RPMI+ 500µl cisplatin/rhodium intercalator solution (10 µM cisplatin (from 1mM stock) +4X Rhodium intercalator (from 500X stock) in RPMI1640 (Ex. 8 mL RPMI+80ul cisplatin 1mM+64ul Rhodium 500X). Incubate for 5 min RT. Quench by adding 5 mL RPMI1640 (10% FBS 1% DNase). Centrifuge 300g 5min. For fixation: resuspend cells in 2 mL RPMI1640+1.6% PFA (8mL PFA 4%+12mL RPMI). Mix well. Incubate for 10 min RT. During incubation, transfer to cryotubes. Finally, centrifuge at 600g 5min RT, aspirate and freeze at -80 °C.

*Cell counting.* 5 tubes at a time were thawed at in a water bath at 37 °C for 2 min, resuspended in 1mL DPBS (0.25 mg/mL DNase, 1% BSA) and left in the water bath for 10 min. Each sample was then well resuspended by pipetting and 10 µl was promptly transferred to a new 1.5 mL tube containing 1 mL PBS before being placed on ice. The concentration of cells in each new tube was found using Cell Countess. Each sample was aliquoted into 3 cryotubes at 3e6 cells. Any leftovers were left in the original tube. All tubes were centrifuged at 300 g for 5 min at RT. Supernatant was aspirated and each pellet was resuspended in 1 mL FBS (10 % DMSO) and placed in a Mr Frosty. The Mr Frosty box was then placed in a -80 °C freezer for at least 2 hours.

*Barcoding.* Five tubes at a time were barcoded. Samples of 1mL 3e6 cells/mL in FBS (10% DMSO) were thawed at 37 °C for 2 min. Each sample was centrifuged at 300 g for 5 min at 4 °C and supernatant was discarded. Each sample was then resuspended in 1 mL DPBS (0.25 mg/mL DNase, 1% BSA) and incubated at 37 °C at 750 rpm in a rotomax. Samples were then centrifuged at 300 g for 5 min at RT. Each sample was resuspended in 1 mL 1x barcode perm buffer and centrifuged at 300 g for 5 min at RT. The samples were washed once more with 1 mL barcode perm buffer. Samples were then resuspended in 800 µl cold 1x barcode perm buffer and placed on ice. Each sample was then resuspended and promptly, 10 µl barcoding solution was resuspended with 100 µl cold 1x barcode perm buffer. The diluted barcoding solution was added to the sample and mixed well and placed in the rotomax and incubated at 750 rpm for 30 min at 37 °C. Samples were then centrifuged and at 300 g for 5 min at RT and supernatant was aspirated. Samples were then resuspended in 1 mL DPBS (0.25mg/mL DNase, 1% BSA) and incubated in the rotomax at 750 rpm for 2 min at 37 °C. The wash was repeated once more. Pellet samples with supernatant discarded were pooled into a 5 mL tube using 100 µl cold FBS (10% DMSO). An additional 1.5 mL was added for a total of 2mL. The pooled barcode samples were mixed well and 1 mL was aliquoted into 2 1.5 mL tubes. The tubes were placed in Mr Frosty and then in a -80 °C freezer for at least 2 hours. Samples 1-20 were additionally barcoded with cisplatin 196, while 21-35 were barcoded with cisplatin 198. Pooled barcoded samples were thawed at 37 °C for 2 min and centrifuged at 1000 g for 2 min at RT. Supernatant was aspirated and the contents of each tube was resuspended in 1 mL 1x barcode perm buffer and transferred to a 15 mL tube. Monoisotopic cisplatin (Pt196 or Pt198) was added to each 15mL tube to yield a concentration of 25 nM before being placed on a rotisserie for 30 min at RT: Each tube was filled with up to 14 mL mark with 1 M Tris to quench the reaction. The tubes were then centrifuged at 1000 g for 8 min. The cells were washed using 14 mL DPBS (0.25mg/mL DNase, 1% BSA) for 10 minutes before being centrifuged at 1000 g for 8 min. Both tubes were then pooled in a total of 8 mL FBS (10% DMSO) and aliquoted into 2 tubes and frozen at -80 °C. 143 µl, roughly 45e6 cells, was set aside to use for titration.

*Antibody conjugation.* Antibody conjugation with 157Gd was performed according to manufacturer instructions. Cadmium labeled antibodies were conjugated according to the cadmium protocol and lanthanides labeled antibodies were conjugated according to the lanthanide protocol. For conjugation of indium, lanthanum, and cerium labeled antibodies, metal salts were dissolved in L-buffer (Maxpar® X8 Antibody Labeling Kit, Fluidigm) to yield a stock solution of 1 000 mM. A diluted solution of 50 mM metal solution was used for antibody conjugation according to the lanthanide protocol.

*Mass Cytometry Staining.* Two tubes containing all barcoded samples were thawed at 37 °C for 2 min. The tubes were centrifuged at 1000 g for 3 min at 4 °C. Supernatant was aspirated and the pellets were resuspended with 1 mL DPBS (0.25mg/mL DNase, 0.5% BSA) (Dwash) and pooled in a 15 mL tube. An additional 1 mL Dwash was used to wash the tubes for remaining cells. The cells were centrifuged at 1000 g for 2 min at 4 °C and supernatant was aspirated. The cells were then incubated in 3 mL Dwash with 1kU/mL heparin for 10 min at RT to block unspecific metal polymer binding. The cells were centrifuged at 1000g for 2 min at 4 °C and supernatant was aspirated. Pellet was vortexed and cells were blocked for 5 min at RT with 1000 fc-block (DPBS, 0.5% BSA, 2 ug/mL of antibody clones 9e9, 93, and 2.4G2, 1 kU/mL heparin). The surface antibody cocktail was added after incubation and cells were incubated at 4 °C overnight on a rotisserie. The next day, 3 mL Dwash was used to wash the cells and cells were then centrifuged at 1000g for 2 min. Cells were washed again with 5 mL Dwash, followed by 5 mL PBS (2 mM EDTA). The cells were transferred to a 50 mL tube using 10 mL PBS (2 mM EDTA). Cells were then fixed for 5 min at RT by adding 1.42 mL 16% PFA. The fixation was quenched by adding 20 mL 1M Tris. The cells were centrifuged at 1000g for 4 min and supernatant was aspirated. Using 4 mL Dwash, the cells were transferred to a 15 mL tube. The cells were centrifuged at 1000g for 2 min and supernatant was discarded. The pellet was vortexed and cells permeabilized using 3 mL -20 °C MeOH that was added dropwise during slow vortexing. The cells were incubated on ice for 10 min. 2 mL Dwash was then added, and the cells were centrifuged at 1000 g for 3 min. The cells were resuspended in 5 mL Dwash and incubated on the bench for 10 min. The cells were then blocked, identical to above before surface staining, before being stained with intracellular antibody cocktail overnight on rotisserie at 4 °C. The next day the cells were washed by resuspending them in 3 mL Dwash and centrifuging at 1000g for 2 min. The cells were washed 2 more times with 5 mL Dwash. The pellet was then vortexed, and cells were resuspended in 10 mL PBS (2 mM EDTA, 0.1uM Iridium intercalator, 2% PFA) and incubated at 4 °C overnight. The next day, 5 mL 1 M Tris was added to quench the fixative. The cells were centrifuged at 1000g for 2 min and supernatant was discarded. The pellet was resuspended in 5 mL 1 M Tris and transferred to a new 5 mL tube. The cells were centrifuged at 1000g for 2 min and supernatant was discarded. The cells were then washed in 5 mL Dwash, followed by a wash in 5 mL PBS (2mM EDTA). The cells were left on Ice until ready to run. Running aliquots of 300 µl were prepared by transferring 300 µl cells to a new 1.5 mL tube, washing with 1 mL MilliQ before centrifuging at 1000g for 2 min and resuspending in 2 mL 1x beads.

*Data Analysis.* Acquired IMD files were converted to FCS-files using default settings in CyTOF (version 6.7.1014, Fluidigm). All remaining analysis was performed in R (version 4.1.3). FCS-files were first subsampled to reduce processing time. Subsampled files were normalized using premessa and opened in FlowJo (version 10.7.1) where XML gating files were made. The XML gating files were used for automatically performing the gating through flowUtils. Gated files were then concatenated before being debarcoded by CATALYST. Debarcoded files were again concatenated into a single data frame with an additional filename column. Spillover was compensated for selected markers by CATALYST using a previously acquired spillover matrix. Compensated data was then transformed using the inverse hyperbolic sine function and a cofactor of 5. For UMAP and clustering, each transformed channel was further divided by the iridium signal. This was done to prevent false signal in low-signal channels, which often correlated with iridium. Transformed data was then used to make a UMAP embedding with uwot and for clustering with PARC through reticulate with a cosine metric. Clusters consisting of debris were removed and remaining clusters were merged and divided in a supervised. The above steps yielded 1 714 280 for the first panel and 5 365 781 events for second panel that were used for further analysis. UMAPs, containing events from all files, were plotted, colored by marker intensity or cluster identity using ggplot2. Heatmaps were made using the heatmap package. A heatmap of mean channel intensity for each cluster was made by first calculating means for each channel in each cluster then range scaling each channel from 0 to 1 (see Supplementary Figure 4). Heatmaps of relative abundance of each cluster in each sample was made by using the clr function on acomp data using the compositions package as described below in the Statistics section. For plotting density UMAPs and UMAPs displaying channel intensity across the groups, the lowest common number of events, 177 548 in panel 1 and 120 162 in panel 2, were used from each group. This allows for easy comparison between groups.
